# The Purinome and the preBötzinger Complex – A Ménage of Unexplored Mechanisms That May Modulate/Shape the Hypoxic Ventilatory Response

**DOI:** 10.3389/fncel.2019.00365

**Published:** 2019-08-21

**Authors:** Robert J. Reklow, Tucaaue S. Alvares, Yong Zhang, Ana P. Miranda Tapia, Vivian Biancardi, Alexis K. Katzell, Sara M. Frangos, Megan A. Hansen, Alexander W. Toohey, Carol E. Cass, James D. Young, Silvia Pagliardini, Detlev Boison, Gregory D. Funk

**Affiliations:** ^1^Department of Physiology, Women and Children’s Health Research Institute, Neuroscience and Mental Health Institute, Faculty of Medicine and Dentistry, University of Alberta, Edmonton, AB, Canada; ^2^Professor Emerita, Department of Oncology, Faculty of Medicine and Dentistry, University of Alberta, Edmonton, AB, Canada; ^3^Department of Neurosurgery, Robert Wood Johnson Medical School and New Jersey Medical School, Rutgers University, New Brunswick, NJ, United States

**Keywords:** hypoxia, P2 receptor, P1 receptor, ectonucleotidase, equilibrative nucleoside transporter, adenosine kinase

## Abstract

Exploration of purinergic signaling in brainstem homeostatic control processes is challenging the traditional view that the biphasic hypoxic ventilatory response, which comprises a rapid initial increase in breathing followed by a slower secondary depression, reflects the interaction between peripheral chemoreceptor-mediated excitation and central inhibition. While controversial, accumulating evidence supports that in addition to peripheral excitation, interactions between central excitatory and inhibitory purinergic mechanisms shape this key homeostatic reflex. The objective of this review is to present our working model of how purinergic signaling modulates the glutamatergic inspiratory synapse in the preBötzinger Complex (key site of inspiratory rhythm generation) to shape the hypoxic ventilatory response. It is based on the perspective that has emerged from decades of analysis of glutamatergic synapses in the hippocampus, where the actions of extracellular ATP are determined by a complex signaling system, the purinome. The purinome involves not only the actions of ATP and adenosine at P2 and P1 receptors, respectively, but diverse families of enzymes and transporters that collectively determine the rate of ATP degradation, adenosine accumulation and adenosine clearance. We summarize current knowledge of the roles played by these different purinergic elements in the hypoxic ventilatory response, often drawing on examples from other brain regions, and look ahead to many unanswered questions and remaining challenges.

## Introduction

The mammalian brain depends on a constant supply of oxygen (O_2_) to meet its energy needs, and a host of adaptive responses have evolved to protect brain O_2_ levels. Prominent among these is the biphasic hypoxic ventilatory response (Mortola, 1996) in which a fall in arterial O_2_ detected at the carotid body chemoreceptors triggers, within the first minute of exposure, an adaptive (Phase 1) increase in breathing. If this increase does not immediately restore arterial O_2_, the brain becomes hypoxic, triggering changes in brain chemistry and a maladaptive secondary hypoxic respiratory depression during which ventilation gradually decreases (4–5 min) to a lower steady-state (Phase 2) level. The secondary depression is especially pronounced in premature mammals, where ventilation falls below baseline and can become life-threatening (Mortola, 1996; Bissonnette, 2000; Moss, 2000).

Mechanistically, the biphasic hypoxic ventilatory response has been viewed for decades as the result of just two interacting processes; an initial peripheral, carotid body-mediated (Phase 1) excitation and a slower, centrally mediated hypoxic respiratory depression to a steady-state (Phase 2) level of breathing (Mortola, 1996; Moss, 2000). The mechanisms underlying this depression are not fully understood, but adenosine is strongly implicated (Mortola, 1996; Bissonnette, 2000; Moss, 2000). The key point is that according to this conventional view of the hypoxic ventilatory response, excitation of breathing during hypoxia derives solely from the peripheral nervous system; i.e., the only contribution of the central nervous system to the hypoxic ventilatory response is depression.

New evidence from key cardiorespiratory control sites is challenging this dogma. In relation to the cardiovascular system, C1 noradrenergic neurons involved in control of heart rate and blood pressure are powerfully excited by hypoxia and this excitation is important for homeostatic control (Guyenet, 2006). In the respiratory network, while the Phase 1 component of the hypoxic ventilatory response is mediated peripherally, our data from rodents strongly suggest that during hypoxia, astrocytes in the preBötzinger Complex (preBötC, critical site for generating breathing rhythm) detect hypoxia and release ATP, which, via P2Y_1_ receptors, excites inspiratory neurons and increases ventilation, thereby attenuating the hypoxic respiratory depression (Gourine et al., 2005; Angelova et al., 2015; Rajani et al., 2017; Sheikhbahaei et al., 2018). Thus, unlike the majority of brain regions where hypoxia has depressant actions, the astroglial network of the preBötC appears to mount an excitatory response that partially counteracts the hypoxic respiratory depression, contributing to a vital homeostatic reflex.

The effects of extracellular ATP (ATPe), however, are not determined solely by its actions on P2 receptors. ATPe is rapidly broken down by ectonucleotidases (e.g., Cd39, CD73) into extracellular adenosine diphosphate (ADPe), adenosine monophosphate (AMPe) and ultimately adenosine (ADOe), a transmitter in its own right that signals via 4 types of P1 receptors, A1, A2A, A2B, and A3 (Haas and Selbach, 2000; Sebastiao and Ribeiro, 2009). Indeed a predominant effect of hypoxia (and ischemia) on brain chemistry is a widespread increase in the concentration of extracellular adenosine (ADOe) (reviewed by Dale and Frenguelli, 2009) that can derive from multiple sources including vesicular release of ATP as a transmitter/cotransmitter that is subsequently degraded, and export of intracellular ADO (ADOi) (reviewed by Latini and Pedata, 2001). In the brain ADOe acts primarily through low affinity A1 and A2A receptors to elicit a host of region-specific effects, largely by modulating glutamatergic transmission. A1 receptor-mediated inhibitory mechanisms, pre- and postsynaptic, are widespread and can be considered neuroprotective (Wei et al., 2011; Boison, 2013a, 2016). A2A receptors are primarily excitatory and engaged in adaptive processes, as heralded by their key role in synaptic plasticity in different brain areas (El Yacoubi et al., 2001, 2009; Pedata et al., 2001; Thauerer et al., 2012; Cunha, 2016; Canas et al., 2018; Lopes et al., 2019). Within the brainstem network that generates and controls breathing, ADOe is largely inhibitory, which in this network is maladaptive; i.e., for the body/brain to recover from hypoxia and restore O_2_ homeostasis, ventilation and cardiac activity must increase. ADOe inhibits breathing most potently in premature and newborn mammals via A1 receptors in the preBötC (Herlenius et al., 1997; Herlenius and Lagercrantz, 1999; Huxtable et al., 2009; Zwicker et al., 2011) and A2A receptor-mediated excitation of brainstem GABAergic neurons (Koos et al., 2001, 2005; Wilson et al., 2004; Mayer et al., 2006). Indeed the inhibitory actions of ADOe on the central respiratory network are strongly implicated in the respiratory depression that is life-threatening in apnea of prematurity (AOP) (Martin and Abu-Shaweesh, 2005; Funk, 2013; Poalillo and Picone, 2013; Burnstock and Dale, 2015), and fatal in sudden infant death syndrome (SIDS) and sudden unexpected death in epilepsy (SUDEP) (Boison, 2013b; Richerson et al., 2016).

**FIGURE 1:**
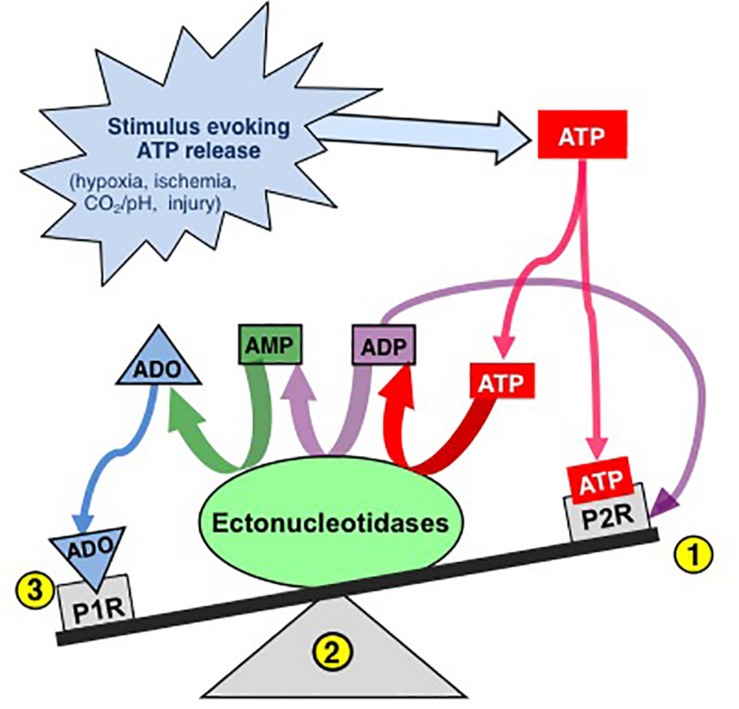
The effects of ATP released into the extracellular space is determined by a balance between actions of ATP at P2 receptors and ADO, its main metabolite, at P1 receptors. Extracellular ATP has two main fates, binding to P2 receptors (1) or degradation by diverse ectonucleotidases (ECTOs) (2) with differential substrate affinities that degrade ATP and its by-products ultimately to adenosine (ADO). Extracellular ADO has three main fates, binding to P1 receptors, transport into the intracellular space or remaining where it is. The balance is dynamic and determined by a complex system referred to as the purinome (see Figure 2) (Modified with permission from Funk, 2013).

**FIGURE 2:**
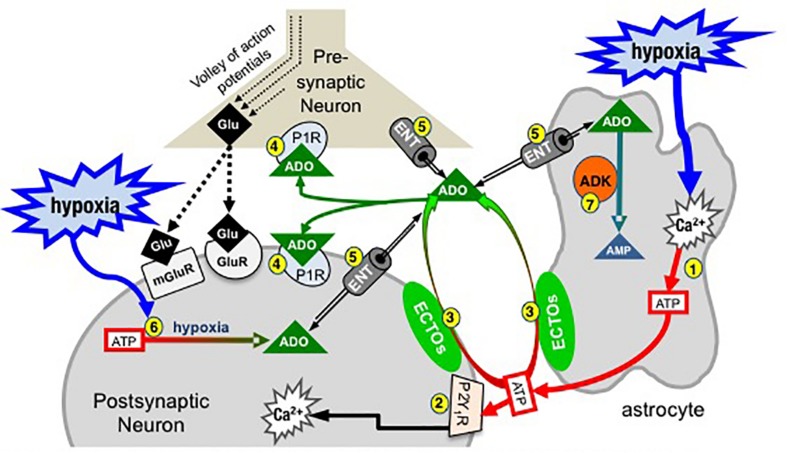
Simplified schematic of the purinome at a glutamatergic, inspiratory, preBötC synapse: multiple factors determine the balance between ATP and ADO signaling. During inspiration, a volley of action potentials in the presynaptic neuron evokes glutamate release that depolarizes the postsynaptic inspiratory neuron via activation of glutamate receptors (ionotropic and metabotropic, mGluR). Hypoxia stimulates astrocytes, via a mitochondrial mechanism (not shown) that evokes increased intracellular Ca^2+^ and vesicular ATP release (1). (2) ATPe acts via neuronal P2 (primarily P2Y_1_) receptors to excite inspiratory neurons and increase ventilation via a process involving increased intracellular Ca^2+^. Extracellular ADO (ADOe) increases through breakdown of ATPe by ectonucleotidases (3) or ENT transport of accumulating ADOi (5). ADO acts pre- and postsynaptically via A1 receptors (or A2 receptors on GABAergic neurons, not shown) to inhibit ventilation (4). The direction of ADO transport via ENTs (5) is dependent on the [ADO] gradient. Hypoxia also causes accumulation of intracellular ADO (ADOi) from ATPi hydrolysis (6). ADK phosphorylates ADO into AMP (7), keeping ADOi low so that ENTs remove ADOe. The cytoplasmic form of ADK, at least in adult brain, is limited to astrocytes so that removal of ADOe becomes an astrocyte dependent process (Modified with permission from Funk, 2013).

Thus, the actions of ATP in the preBötC are likely determined by the balance between the excitatory actions of ATP (and ADP) at P2 receptors and the inhibitory actions of its main metabolite, ADO, at P1 receptors (Figure 1). Indeed, this balance, which is controlled by a complex signaling system referred to as the *purinome* (Volonte and D’Ambrosi, 2009; Figure 2), is emerging as important in determining the degree of hypoxic respiratory depression (Funk, 2013; Angelova et al., 2015; Gourine and Funk, 2017; Rajani et al., 2017; Funk and Gourine, 2018a). The purinome includes: ATPe and P2 receptors (1,2 in Figure 2), ADOe and P1 receptors (4); ectonucleotidases that degrade ATP into ADO (3); equilibrative and concentrative nucleoside transporters (ENTs and CNTs, respectively) (King et al., 2006; Parkinson et al., 2011; Young et al., 2013) that move ADO across membranes down (ENTs) or against (CNTs) the ADO concentration gradient (5); and intracellular metabolic enzymes such as adenosine kinase (ADK) (Boison, 2013b) that keep ADOi levels low and control the direction of ADO transport by ENTs (7) (Boison, 2013b). Astrocytes also contribute through their ability to release and respond to ATP and ADO, degrade ATP and remove ADOe. This integrated view of the purinome has influenced epilepsy researchers in the development of novel strategies for manipulating endogenous levels of ADOe to combat seizures (Boison, 2012b; Richerson et al., 2016). Previous work exploring the contribution of purinergic signaling to the hypoxic ventilatory response has been neurocentric and focused primarily on the actions of ADO at P1 receptors. Thus, contrary to the long-held view that the biphasic hypoxic ventilatory response is due to two competing processes, we propose at least three processes, a peripheral carotid body mediated excitation that underlies Phase 1, as well as central excitatory and inhibitory processes that interact to determine the time course and magnitude of the secondary depression; we also propose a key role for glia in this central excitation. Here, we first discuss the clinical significance of understanding the hypoxic ventilatory response. We then present our working hypothesis of the significance of purinergic signaling in the hypoxic ventilatory response from the broader perspective of the purinome, summarize what is known about the roles played by each component of the purinome in this response and highlight some of the important challenges/questions that remain. Our purpose is not to provide an exhaustive review of all purinergic mechanisms and their influence on information processing in the CNS, but to focus on those most relevant to understanding purinergic signaling in the preBötC and its contribution to the hypoxic ventilatory response. We draw from the insights about purinergic signaling that come from other systems, especially the Schaffer collateral-CA1 pyramidal neuron synapse in the hippocampus, and consider potential implications of identified mechanisms in the context of what is known in respiratory control. For those interested in the control of breathing, the goal is familiarity with the complexities of purinergic systems and potential implications for respiratory control in health and disease. For those with expertise in purinergic signaling, the goal is an appreciation of the unique opportunities for advancing understanding of purinergic signaling that might come with analysis of the central neuro-glial networks that control breathing [the contribution of purinergic signaling to hypoxia sensing in the carotid body is reviewed elsewhere (Lahiri et al., 2007; Conde et al., 2017; Leonard et al., 2018; Nurse et al., 2018)].

## Clinical Significance of the Hypoxic Ventilatory Response

Hypoxia and the secondary hypoxic respiratory depression are most severe and life threatening in infants who are born prematurely. This reflects that the brain circuits responsible for the generation and control of ventilation are immature and produce a breathing pattern that is interrupted by frequent apneas (periods where breathing stops; apnea of prematurity). Thus, a potentially fatal positive feedback loop can develop in which an apnea causes hypoxia, hypoxia evokes the hypoxic ventilatory response that features a strong secondary respiratory depression that can exacerbate the hypoxia…and so on. Apnea of prematurity affects ∼1% of all births in Canada; ∼3000 babies/yr (Statistics Canada., 2014). Risk decreases dramatically with gestational age; ∼15% of infants are affected at 32–33 weeks gestational age but nearly 100% at < 29 weeks (Poalillo and Picone, 2013). The mechanisms underlying the hypoxic respiratory depression and the greater depression in prematurity are not fully understood. The literature is confusing because the hypoxic respiratory depression varies greatly between species, changes developmentally, and likely involves multiple mechanisms. It also depends on whether the experimental apparatus used to deliver the hypoxic gas can deliver a rapid, step change in oxygen. The hypoxia-evoked, Phase 1 increase in ventilation peaks within the 1st min. If the transition from normoxia to hypoxia produced by the gas delivery system is too slow, the hypoxic stimulus will peak after secondary depressive mechanisms have been activated. The result is that the magnitude of both the Phase 1 increase in ventilation and the secondary depression will be underestimated. Nevertheless, it is clear that while the secondary depression is not entirely due to ADOe, ADOe plays a significant role (Martin and Abu-Shaweesh, 2005; Funk, 2013; Poalillo and Picone, 2013; Burnstock and Dale, 2015). Blockade of the respiratory depression by ADO receptor antagonists in a host of species suggests a causative role for ADOe (Martin and Abu-Shaweesh, 2005; Funk, 2013; Poalillo and Picone, 2013; Burnstock and Dale, 2015). Virtually all infants born prematurely who have apnea are treated with methylxanthines, purine based respiratory stimulants that antagonize the inhibitory actions of ADO (Schmidt et al., 2007, 2012). At higher doses that may be relevant clinically, methylxanthines may also inhibit phosphodiesterase activity (preventing cAMP breakdown) and voltage-gated calcium channels (Comer et al., 2001); i.e., the actions of caffeine may not all be mediated by P1 receptors.

Caffeine is the preferred methylxanthine for treatment of apnea of prematurity, due to its better safety profile, and efficacy (Shrestha and Jawa, 2017). However, there is still a need for alternate treatment strategies. First, ∼20% of apnea of prematurity patients do not respond to caffeine (Schmidt et al., 2007, 2012). On average such infants will spend an extra week on ventilator support and face greater rates of lung pathology, cognitive delay and cerebral palsy (Schmidt et al., 2007, 2012). Second, while generally a very safe drug, acute side effects include tachycardia, hypertension and tremors. In addition, high concentrations (which are more effective at reducing apneas) in preterm infants increase the incidence of cerebellar hemorrhage 2 years later, are associated with significant changes in motor performance (McPherson et al., 2015) and status epilepticus (Boison, 2011; Shrestha and Jawa, 2017). There is also the potential for long-term effects on sleep (Montandon et al., 2009). Hypoxia and hypoxic respiratory depression are also implicated in SUDEP, the sudden, unexplained death of persons with epilepsy (Boison, 2013b; Richerson et al., 2016; Vilella et al., 2018). SUDEP is a catastrophic event for which all persons with epilepsy are at risk; most deaths occur in middle age but it can happen at any age. The risk for SUDEP is not trivial. A cohort of children with epilepsy was observed for 40 years; SUDEP occurred in 9% of all patients and accounted for 38% of all deaths (Sillanpaa and Shinnar, 2010; Boison, 2012a). Respiratory arrest appears to be the major cause for SUDEP (Devinsky, 2011); a leading hypothesis, the ADO hypothesis of SUDEP, proposes that hypoxia and accumulation of ADOe during a seizure causes a fatal depression of the brainstem respiratory networks (Shen et al., 2010). No preventative strategies exist for SUDEP.

Intermittent hypoxia is also common in various forms of sleep disordered breathing in which a combination of a collapsible airway, high arousability, and high loop gain in chemosensory control systems give rise to cyclic apneas (Dempsey et al., 2012) and a dramatic increase in the risk of cardiovascular disease (Young et al., 1993; Young and Peppard, 2000; Leung and Bradley, 2001; Punjabi, 2008). While the hypoxic ventilatory response and hypoxic respiratory depression are not directly implicated in the cyclic apneas of obstructive sleep apnea, understanding all chemosensory systems, including the novel central hypoxia sensing mechanism under discussion here, is essential to resolve how central and peripheral chemosensory systems interact to cause high loop gain and cyclic apneas that are common in sleep disordered breathing (Dempsey et al., 2010, 2012; Dempsey and Smith, 2019).

Efforts to understand purinergic signaling in the hypoxic ventilatory response have largely focused on the inhibitory actions of ADO at P1 receptors, primarily because ADO is so strongly implicated in the profound hypoxic depression in apnea of prematurity (Martin and Abu-Shaweesh, 2005; Funk, 2013; Poalillo and Picone, 2013; Burnstock and Dale, 2015). Given the emerging picture that the balance between ATP and ADO signaling in the preBötC is important in determining the degree of respiratory depression during the hypoxic ventilatory response, this focus on ADO receptors needs to expand to other components of the purinome. Understanding how the various components of the purinome affect the balance between ATP and ADO signaling is key to manipulating purinergic signaling to modulate breathing. Indeed, the therapeutic potential of the purinome lies in the diversity of its ATP and ADO receptors, transporters, and enzymes. This is fertile ground that has led to clinical drug trials for cardiac arrhythmias, pain, thrombosis, Parkinson’s disease, psoriasis, dry eye, cystic fibrosis, glaucoma and cancer (Pacak et al., 2002; Koles et al., 2005; Burnstock, 2006; Jacobson et al., 2012; Boison, 2013b).

## Working Model of Purinergic Signaling in the preBötC Inspiratory Synapse During Hypoxia

We first provide a brief summary of our working model of how the various components of the purinome in the preBötC might shape the hypoxic ventilatory response; note that not all of the indicated pathways have been demonstrated. This summary is followed by a detailed discussion of the data supporting involvement of purinergic signaling in each step of the proposed model that focuses on the types of preparations from which data were derived (culture, *in vitro*, or *in vivo* anesthetized/paralyzed/freely moving). When relevant data are not available from analysis of the respiratory network, we draw on insights made from analysis of glutamatergic synapses in other brain regions, in particular the hippocampus, where the modulation of glutamatergic signaling by purines (and all components of the purinome) is more completely understood.

At the core of the model (Figure 2) are three preBötC cells including an astrocyte, a presynaptic inspiratory glutamatergic neuron, and a postsynaptic, inspiratory glutamatergic neuron. During inspiration a volley of action potentials arrives at the presynaptic terminal, triggers the release of glutamate that acts at ionotropic (primarily AMPA) and metabotropic glutamate receptors and evokes an inspiratory burst. Modulation of rhythm by ATP and ADO during hypoxia is hypothesized to occur through modulation of excitability at multiple synapses like this one between key inspiratory, preBötC neurons that generate rhythm based on their excitatory connections with each other (Del Negro et al., 2018). During hypoxia, astrocytes in the preBötC respond with an increase in intracellular Ca^2+^ and vesicular release of ATP (1). ATPe has two main fates. It binds to P2, primarily P2Y_1_, receptors on inspiratory neurons causing excitation and increased inspiratory frequency, at least in part by activating G_*q*_ proteins and increasing intracellular Ca^2+^ (Rajani et al., 2017) (2). ATPe, once released into the extracellular space, also immediately begins to undergo degradation by ectonucleotidases (3), producing extracellular ADPe (which is excitatory at P2Y_1_ receptors), AMPe and finally ADOe, which binds to pre- and postsynaptic A1 receptors that inhibit inspiratory rhythm (4). This sets up the very dynamic interaction between the excitatory actions of ATPe (and ADPe) at P2 receptors (2) and inhibitory actions of ADOe at A1 receptors (4). The dynamics will be determined by all elements of the purinome that likely vary between brain regions, with development and also between the same brain region in different species (see discussion below comparing rat vs. mice (Zwicker et al., 2011). Excitation will be determined by the amount of ATP released, local P2 receptor expression patterns and levels, their downstream signaling cascades, as well as the expression level and local complement of ectonucleotidases that determine the rate of ATP removal. In addition, because different ectonucleotidases have different substrate affinities and reaction products with some preferentially producing ADP (which is excitatory via P2Y_1_ receptors) while others preferentially produce ADO, the mixture of agonists that develops following ATP release depends on the local complement of ectonucleotidases. Inhibition will be determined by local P1 receptor expression patterns and levels, their downstream signaling cascades and the local concentration of ADOe. ADOe accumulates from ectonucleotidase-mediated degradation of adenine nucleotides but it can also come from ENT-mediated transport of ADOi (5) if the concentration of ADO accumulating inside cells from ATP hydrolysis (6) during hypoxia exceeds ADOe. Importantly, the availability of ADOe and hence the level of neuronal ADO receptor activation is largely under the control of ADK (7), expressed in astrocytes (at least in adults). Intracellular phosphorylation of ADO into AMP in astrocytes keeps ADOi levels sufficiently low to drive the influx of ADOe into astrocytes via ENT facilitated transport. Thereby, astrocytes assume a role as a metabolic sink for ADOe and for the termination of ADO receptor activation. The main points of Figure 2 are first that the balance between P2 and P1 receptor signaling is very dynamic and determined by multiple factors, many of which remain to be examined for their impact on the hypoxic ventilatory response. Second, in contrast to the long-held view that the biphasic hypoxic ventilatory response results from just two interacting processes [an initial peripheral, carotid body-mediated (Phase 1) excitation and a slower, centrally mediated hypoxic respiratory depression (Mortola, 1996; Moss, 2000)], we propose that following the Phase 1 increase, central purinergic mechanisms, excitatory and inhibitory, interact in the preBötC and perhaps elsewhere to shape the remainder of the hypoxic ventilatory response.

While the initial increase indeed appears to be mediated by peripheral chemoreceptors, whether a central excitatory component helps shape the hypoxic ventilatory response during Phase 2 remains controversial and readers are referred to a recent Cross-Talk debate in the Journal of Physiology for a detailed discussion (Funk and Gourine, 2018a, b; Teppema, 2018a, b). Several points of consensus came from that Cross-Talk. First, there was general agreement that gradual recovery, or lack of recovery, of an excitatory component to the hypoxic ventilatory response following carotid body denervation is unlikely to be informative as the same data could be interpreted to support or refute either position. For example, while the frequently observed loss of an hypoxia-evoked increase in ventilation after carotid body denervation is compelling evidence that there is no central contribution to the hypoxia-induced increase in ventilation, there is an alternate interpretation. The lack of response could result from the loss of a tonic, non-chemosensory related carotid body input that is necessary for central hypoxia sensing mechanisms to be expressed. Indeed, unanesthetized dogs and goats with separately perfused, normoxic carotid bodies respond to central hypoxia with a slow onset increase in ventilation that is lost once the normoxic carotid bodies are denervated (Daristotle et al., 1991; Curran et al., 2000). Conversely, gradual recovery of an excitatory ventilatory response to hypoxia in carotid body-denervated animals is often cited as strong evidence of a central contribution, but this recovered response could also be the result of plasticity. Indeed, carotid body denervation triggers considerable plasticity in peripheral and central neural networks involved in the control of breathing (Teppema and Dahan, 2010). Second, the need for reduced preparations to delineate cellular/ionic/molecular mechanisms of O_2_ sensing was acknowledged, but so was the importance of acknowledging the limitations of data derived from such preparations. Finally, there was consensus that the real arbiter of the physiological relevance of central hypoxia sensing mechanisms is what happens in unanesthetized animals with intact carotid bodies when putative central oxygen sensing mechanisms are selectively (and reversibly) perturbed. Progress toward this goal, via the application of viral approaches to selectively (but not yet reversibly) manipulate central purinergic and glial signaling mechanisms *in vivo*, is summarized below.

## Components of the Purinome and their Role(s) in the Hypoxic Ventilatory Response

### Role for ATP and Astrocytes

Hypoxia-evoked ATP release of unknown origin was first detected using ATP sensors placed on the ventral medullary surface of anesthetized rats (Gourine et al., 2005; Angelova et al., 2015; Rajani et al., 2017). Hypoxia-induced increases in intracellular Ca^2+^ fluorescence of cortical astrocytes in anesthetized rats suggest that astrocytes are directly sensitive to hypoxia. Direct sensitivity was confirmed with the demonstration that cultured astrocytes respond to hypoxia with an increase in mitochondrial reactive oxygen species that activate PLC, causing an increase in intracellular Ca^2+^ and exocytotic release of ATP (not shown in Figure 2); ATP release was shown directly using TIRF (total internal reflection fluorescence) imaging to reveal hypoxia-induced disappearance of ATP-containing vesicles from the intracellular surface of astrocyte membranes (Angelova et al., 2015).

Astrocytic ATP release was also shown indirectly in awake, carotid body-intact animals using viral approaches to block astroglial vesicular release mechanisms (injection of adenoviral vectors that expressed either the light chain tetanus toxin or the dominant-negative SNARE protein in astrocytes) or increase ATP degradation (injection of lentiviral vector that increased ectonucleotidase expression on all cells) at the level of the preBötC; both treatments consistently reduced the hypoxic ventilatory response (Angelova et al., 2015; Sheikhbahaei et al., 2018). These treatments in anesthetized, carotid body intact or carotid body denervated rats similarly decreased the hypoxic ventilatory response (Angelova et al., 2015; Rajani et al., 2017).

These data make a strong case for an ATP-mediated, excitatory contribution to the hypoxic ventilatory response. A caveat remains regarding the case for an astrocytic contribution. The recent demonstrations that disruption of vesicular release mechanisms in astrocytes using the same viral tools attenuates the hypercapnic ventilatory response and exercise capacity as well as the hypoxic ventilatory response (Marina et al., 2017; Sheikhbahaei et al., 2018) have raised the concern that viral injection disrupts baseline astrocyte functions and impairs preBötC excitability. We consider this unlikely because control viruses were without effect on the hypoxic ventilatory response, the hypercapnic ventilatory response and exercise capacity. Thus, while it will be important to demonstrate that the viral tools used to disrupt astrocytic signaling *in vivo* do not globally impair preBötC responsiveness, we propose that the attenuation of respiratory responses to elevated ventilatory drive or metabolic demand following block of vesicular release mechanisms in preBötC astrocytes supports that astrocytes act as brain metabolic sensors (Marina et al., 2017).

### P2 Receptors

ATP acts through seven subtypes of ionotropic P2X and eight subtypes of metabotropic P2Y receptors (Abbracchio et al., 2009; Burnstock, 2015; Burnstock and Dale, 2015). Our focus is on P2Y_1_ receptors because they are exclusively responsible for the marked frequency increase evoked by ATP in the preBötC in medullary slices that generate inspiratory-related rhythm *in vitro*. However, P2Y_1_ receptor effects are not always excitatory and vary between brain regions. For example, in hippocampus P2Y_1_ receptor activation reduces glutamate release (Rodrigues et al., 2005), but also excites inhibitory interneurons (Kawamura et al., 2004). Lamina IX spinal cord neurons are also directly excited by P2Y_1_ receptor activation (Aoyama et al., 2010). In the preBötC, MRS 2179 and MRS 2279 (P2Y_1_ receptor antagonists) completely block the network response evoked by exogenous ATP *in vitro*, while PPADS and Suramin (general P2 antagonists with low affinity for P2Y_1_ receptors) and TNP-ATP (P2X_1__,__3_ antagonist) are without effect (Lorier et al., 2007; Huxtable et al., 2009, 2010). *In vivo*, bathing the ventral medullary surface of anesthetized rats with PPADS (10 μM) or unilaterally injecting the P2Y_1_ antagonist MRS 2279 into the preBötC of paralyzed rats reduces the hypoxic ventilatory response, indicating that P2Y_1_ receptors, and possibly other P2 receptors, contribute to the central, hypoxia-mediated increase in ventilation (Gourine et al., 2005; Angelova et al., 2015; Rajani et al., 2017). Potentiation of P2Y_1_ receptor signaling may therefore be one approach through which respiratory activity could be enhanced to counteract respiratory depression.

These data add to a growing body of evidence that the preBötC is unique. Not only is it key for inspiratory rhythm generation (Feldman and Del Negro, 2006), generation of sighs (Li et al., 2016) and coordinating multiple orofacial behaviors (Kleinfeld et al., 2014a, b), it also mounts an excitatory response to hypoxia (Angelova et al., 2015; Gourine and Funk, 2017; Rajani et al., 2017). An important remaining task is to define 2nd messenger cascades, ionic mechanisms and neuronal phenotype(s) that underlie the ATP excitation of the preBötC that occurs during hypoxia. P2Y_1_ receptors conventionally signal via the G_*q*_ cascade (Abbracchio et al., 2006), but the cascade activated in the preBötC has not been identified. Candidate ion channels, i.e., those that affect preBötC rhythm and are also modulated by P2Y_1_ receptors, include TASK, KCNQ, L-, P-, Q-, and N-type voltage-gated Ca^2+^ channels, BK and SK, and I_*CAN*_ (Ca^2+^-activated non-selective cation channels) (Rajani et al., 2016). A key question is whether the subgroup of preBötC neurons that respond to P2Y_1_ receptor activation and mediate the increase in network frequency are excitatory (i.e., glutamatergic) or inhibitory (GABA- or glycinergic). The model in Figure 2 depicts glutamatergic neurons as responsible but recent data indicate that preBötC frequency can be more dramatically increased via the activation of inhibitory preBötC neurons (Baertsch et al., 2018).

### Ectonucleotidases

There are seven extracellular ectonucleotidase isoforms in the brain grouped into four families: E-NTPDase-1-3; E-NPP1 and 3; tissue non-specific alkaline phosphatase (TNAP); and ecto-5′-nucleotidase (Langer et al., 2008; Abbracchio et al., 2009). This diversity, combined with isoform diversity in substrate and end-product preferences and differential distribution in the brain underlies significant clinical potential and ongoing efforts to produce selective ectonucleotidase agonists/antagonists (al-Rashida and Iqbal, 2014). There is little doubt that ectonucleotidases help determine the balance between ATP and ADO signaling in the preBötC (Funk et al., 2008; Huxtable et al., 2009; Zwicker et al., 2011). However, that most ectonucleotidase inhibitors lack specificity and have off-target actions that interfere with respiratory network function means that most evidence is indirect. Evidence that ectonucleotidases affect preBötC network responses to ATP derives primarily from analysis of rhythmically active medullary slices *in vitro* and includes that: (i) hydrolysis-resistant ATP analogs, such as ATPγS (Lorier et al., 2007) and MRS 2365 (Huxtable et al., 2009) evoke greater frequency responses than ATP; (ii) frequency responses evoked by injection of ATP into the preBötC of rat and especially mouse slices are enhanced by the A1 receptor antagonist, DPCPX (Huxtable et al., 2009; Zwicker et al., 2011); (iii) diffusion of ATP through the preBötC is enhanced in tissue with reduced ectonucleotidase activity (Huxtable et al., 2009); (iv) ATP degradation, measured as the rate of phosphate produced by preBötC tissue punches incubated with ATP, is greatly slowed by the ectonucleotidase inhibitor POM1 (Huxtable et al., 2009); (v) ATP applied to the saline solution bathing rhythmically active slices has very poor access into the tissue; i.e., for an ATP sensor placed just above the slice surface to record the same ATP signal as when the same sensor is placed just 200 μm below the slice surface in the preBötC, the concentration of ATP in the bath must be raised 100-fold, suggesting limited diffusion but more likely significant ATP degradation (Funk et al., 2008).

**FIGURE 3:**
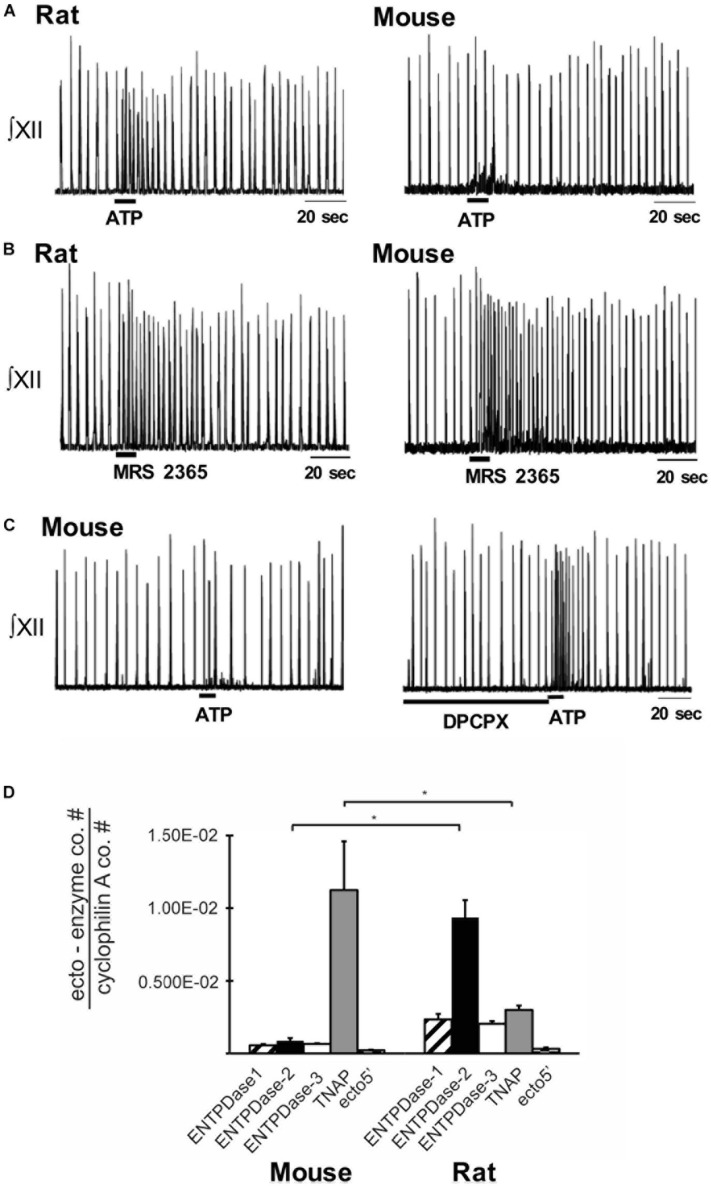
Differential balance between the actions of ATP and ADO in rhythmically-active preBötC-containing slices from neonatal rat and mouse. Integrated XII nerve recordings (∫XII) from rhythmic slices of rat and mice showing baseline inspiratory-related rhythm *in vitro* and responses to local injection of ATP **(A)** or the P2Y_1_ receptor agonist, MRS-2365, into the preBötC **(B)**. **(C)** Response of a mouse slice to ATP under control conditions and after preBötC injection of the A1 receptor antagonist DPCPX. **(D)** Real-time PCR analysis showing the percentage contribution of each ectonucleotidase isoform to the total ectonucleotidase mRNA extracted from mouse and rat preBötC punches. Error bars indicate SEM. ^∗^Significant difference between the compared columns. Reproduced with permission from Zwicker et al. (2011).

Indirect evidence that differential ectonucleotidase expression is an important factor in shaping network response dynamics to ATP comes from comparing the responses evoked by injecting ATP into the preBötC of slices from rats and mice and correlating these data with real-time PCR data quantifying the relative expression patterns of ectonucleotidase transcripts in the preBötC of the two species. When applied to the preBötC of Wistar or Sprague-Dawley rats, a brief injection of ATP (10 s) evokes a two- to four-fold increase in frequency that peaks in the first 20–30 s and then falls, typically below baseline between 30 and 60 s postinjection before returning to baseline. In mice, however, the same ATP injection either has no effect or a much smaller effect on frequency (Figure 3A) (Zwicker et al., 2011). Importantly, the response of the mouse preBötC to ATP is the same as the rat if A1 ADO receptors are first blocked (Figure 3C). Rat and mouse responses to the selective P2Y_1_ receptor agonist MRS 2365 are also very similar (Figure 3B). Thus, both networks share a common P2 receptor-mediated excitation (Zwicker et al., 2011). In mouse, however, it appears that ATP is degraded so quickly to ADO that the ATP excitation and ADO inhibition almost cancel each other out. That the complement of ectonucleotidases might be a factor in this differential ATP sensitivity is supported by the real-time PCR analysis of mRNA extracted from preBötC. The protein encoded by the dominant ectonucleotidase transcript in mice, TNAP (tissue non-specific alkaline phosphatase), which comprises 80% of total ectonucleotidase mRNA, converts ATPe directly to ADOe. In rats, however, the dominant isoform is ENTPDase 2 (its transcript comprises 55% of total ectonucleotidase mRNA, which has a much higher affinity for ATP than ADP so will preferentially produce ADP, an agonist at excitatory P2Y_1_ receptors (Figure 3D). What remains is to determine protein expression (rather than mRNA) and enzyme activities in these different species as well as between different brain regions. The development of inhibitors that are selective for the different ectonucleotidase isoforms that are also devoid of off-target actions will open the door to answering key questions about the significance and therapeutic potential of manipulating ectonucleotidase activities.

### Adenosine and Adenosine (P1) Receptors

ADO actions are mediated via four subtypes of G-protein coupled, P1 receptors. These are the A1 and A2A high-affinity subtypes and the A2B, and A3 low-affinity subtypes (Sebastiao and Ribeiro, 2009; Burnstock et al., 2011). While dogma holds that A1 and A3 receptors act through Gi/Go to inhibit, while A2A and A2B receptors act through Gs to stimulate, the classical adenylate cyclase – cAMP – protein kinase A signaling pathway (Fredholm et al., 2001; Thauerer et al., 2012), in the brain cAMP does not appear to be the main transducing system operated by A1 or A2A receptors (Cunha, 2016). P1 receptors may also signal through phospholipase C, Ca^2+^ and mitogen-activated protein kinases (MAPKs) (van Calker et al., 1979; Linden, 1991; Linden et al., 1991; Abbracchio et al., 1995; Fredholm et al., 2001; Schulte and Fredholm, 2003; Tomaselli et al., 2008). A2A receptors in the striatum can also couple to G_*olf*_ rather than Gs (Kull et al., 2000). All receptor subtypes are present in the CNS, but the high-affinity A1 and A2A subtypes are functionally the most important (Fredholm et al., 2001; Thauerer et al., 2012).

Measurements of ADOe concentrations made using multiple methods, all with limitations, underlie estimates of basal ADOe levels between 30 and 250 nM (Latini and Pedata, 2001). During hypoxia, local or global ischemia, or traumatic brain injury the concentration of ADOe is estimated to increase up to 100-fold into the μM range (3–30 μM) (Dunwiddie and Masino, 2001; Pedata et al., 2001; Wei et al., 2011). When considered in relation to the binding affinity of the different subtypes for ADO (calculated from *in vitro* binding studies; 3–30 nM for A1, 1–20 nM for A2A, 5 ± 20 μM for A2B and > 1 μM for A3 (Fredholm et al., 1994), ADOe concentration data suggest that basal levels of ADOe are sufficient to tonically activate A1 and A2A receptors. However, basal ADOe transmission appears to primarily to involve A1 receptors (Lopes et al., 2019). Lower affinity A2B and A3 receptors may be activated during hypoxia/ischemia/brain injury when ADOe concentrations increase pathologically (Latini and Pedata, 2001; Pedata et al., 2001).

#### A1 Receptors

A1 receptors are distributed throughout the body with the highest levels of expression in the brain, especially the cortex, hippocampus, cerebellum and dorsal horn of the spinal cord (Mahan et al., 1991; Dixon et al., 1996; Fredholm et al., 2000; Wei et al., 2011), A1 receptor expression is also high in brainstem regions essential for respiratory control (Reppert et al., 1991). As reviewed by Gomes et al. (2011), neuronal A1 receptors are located pre-, post- and extrasynaptically. A1 receptors are highly expressed in presynaptic terminals, especially at excitatory synapses (glutamatergic, cholinergic, serotonergic) (Gomes et al., 2011; Thauerer et al., 2012), where their activation generally inhibits neurotransmitter release (Yawo and Chuhma, 1993; Wu and Saggau, 1994; Gomes et al., 2011), reducing evoked PSPs and the frequency of mEPSPs. The main mechanisms underlying this inhibition include inhibition of voltage-gated Ca^2+^ channels and reduced sensitivity of the vesicular release apparatus to Ca^2+^ (Silinsky, 1984; Scanziani et al., 1992; Scholz and Miller, 1992; Ambrosio et al., 1997). Postsynaptic A1 receptors are primarily located in the postsynaptic density (Rebola et al., 2003) and their activation reduces the responses of hippocampal neurons to excitatory inputs via the inhibition of N-type Ca^2+^ channels, and NMDA receptor-mediated (but not AMPA receptor-mediated) synaptic inputs (de Mendonca et al., 1995).

At the level of the brainstem respiratory network, particularly the preBötC, A1 receptor actions appear dominant (Funk, 2013). A1 receptor antagonists increase basal frequency *in vitro* and *in vivo* and block the inhibitory actions of ADO on respiratory rhythm (Kawai et al., 1995; Schmidt et al., 1995; Kobayashi et al., 2001; Koos et al., 2001, 2005; Wang et al., 2005; Huxtable et al., 2009; Zwicker et al., 2011). In contrast, A2A and A2B receptor agonists have no effect on basal activity of rhythmic medullary slice (Mironov et al., 1999) or brainstem spinal cord preparations from mice (Brockhaus and Ballanyi, 2000), nor on the ADO-mediated inhibition of mouse slices (Mironov et al., 1999). As is well-documented in hippocampus and striatum, A1 receptor activation appears to modulate respiratory neuron and network excitability via pre- and postsynaptic mechanisms, but effects differ between subtypes of respiratory neuron, species and with development. Presynaptic A1 receptor-mediated inhibition of synaptic transmission has only been directly shown with mini PSC analysis in phrenic motoneurons in neonatal rats (Dong and Feldman, 1995). However, presynaptic A1 receptor mediated inhibition of inspiratory, excitatory and inhibitory inputs to rat XII motoneurons *in vitro* (Bellingham and Berger, 1994), mouse inspiratory neurons *in vitro* (Herlenius et al., 1997; Herlenius and Lagercrantz, 1999; Mironov et al., 1999) and cat stage 2 expiratory neurons *in vivo* (Schmidt et al., 1995) is inferred from A1 agonist-mediated reductions in respiratory (excitatory and inhibitory), evoked or spontaneous synaptic inputs (Brockhaus and Ballanyi, 2000). A1 receptor-mediated presynaptic inhibition may also be input- or neuron-specific because the inspiratory synaptic inputs to some inspiratory neurons in the brainstem spinal cord preparation are not unaffected by ADO while excitatory PSPs arriving during expiration are inhibited (Brockhaus and Ballanyi, 2000). Detailed analysis of the effects of ADO and A1 receptor agonists on the frequency and amplitude of miniPSCs in the different subtypes of respiratory neurons is essential to establish which synapses are under presynaptic A1 receptor control.

Postsynaptically, A1 receptor activation hyperpolarizes stage 2 expiratory neurons in adult cat *in vivo* via activation of postsynaptic conductance (likely a K^+^ conductance) that decreases input resistance (Schmidt et al., 1995). Similarly, in the brainstem spinal cord preparation A1 receptor activation hyperpolarizes expiratory neurons and reduces input resistance (effects that persist in TTX), suggesting activation of a postsynaptic K^+^ conductance (Herlenius and Lagercrantz, 1999). The membrane potential and input resistance of biphasic expiratory (Herlenius and Lagercrantz, 1999) and inspiratory neurons are unaffected by ADO in the absence (Brockhaus and Ballanyi, 2000) (or presence) of TTX (Herlenius and Lagercrantz, 1999). In inspiratory preBötC neurons of rhythmic mouse slices, A1 receptors inhibit intracellular cAMP production, which results in K_*ATP*_ activation and membrane hyperpolarization (Mironov et al., 1999). Similarly, A1 receptor-mediated hyperpolarization of inspiratory neurons in preBötC island preparations from mice is associated with significant reductions in neuronal input resistance (Vandam et al., 2008). Developmental and species differences must also be taken into account. For example, the A1 receptor-mediated inhibition of inspiratory rhythm in the rhythmic slice or brainstem spinal-cord preparation of rat is gone by postnatal day 2–3 (Herlenius et al., 1997, 2002; Huxtable et al., 2009), while in mouse it persists until at least 2 weeks of age (Funk and Reklow, unpublished observations).

Postsynaptic A1 receptors are also located at extrasynaptic sites (Tetzlaff et al., 1987). Their activation in hippocampal neurons enhances background K^+^ currents and causes neuronal hyperpolarization (Greene and Haas, 1991). Basal ADOe tone in the CSF should be sufficient to activate these extrasynaptic receptors and contribute to a tonic, ADO-mediated inhibitory drive. The observation that global inhibition of ADO or A1Rs enhances respiratory rhythm in anesthetized adult cats (Schmidt et al., 1995), unanesthetized fetal sheep (Koos et al., 2001), lambs (Koos et al., 2005), rats *in vivo*, neonatal rats *in vitro* (Kawai et al., 1995; Wang et al., 2005), as well as fetal breathing movements in embryonic rats *in vivo* (Kobayashi et al., 2001) and fictive breathing *in vitro* (Huxtable et al., 2009), suggests that the respiratory network is also under tonic A1 receptor inhibitory control. However, whether this tone reflects activation of synaptic or extrasynaptic receptors is not known. Whether A1 receptors on astrocytes and microglia (van Calker and Biber, 2005; Haselkorn et al., 2010) influence respiratory network function also remains to be determined.

#### A2A Receptors

A2A receptors are expressed widely throughout the CNS, but more variably compared to A1 receptors. Expression levels are greatest in the olfactory tubercle and on medium spiny neurons of the striatum (Svenningsson et al., 1997a, b; Rosin et al., 1998). The majority of A2A receptors are located in synapses. In the striatum this includes significant pre-synaptic A2A receptor expression, but the majority of A2A receptors are postsynaptic (Rebola et al., 2005). While A2A expression levels in most other brain regions [including the cortex and hippocampus that show the highest levels of A1R expression (Dixon et al., 1996; Svenningsson et al., 1997a)] are substantially lower than in the striatum, levels are still sufficient to modulate neuronal excitability. For example, activation of presynaptic A2A receptors facilitates glutamatergic transmission in hippocampal CA1 neurons by blocking the inhibitory actions of presynaptic A1 receptors on glutamate release (Lopes et al., 1999, 2002). Postsynaptic A2A receptor mechanisms include potentiation of synaptic, but not extrasynaptic, NMDA receptor responses in hippocampal CA1 neurons (Mouro et al., 2018) and inhibition of the slow afterhyperpolarization in pyramidal neurons of the basolateral amygdala (Rau et al., 2015). As reviewed by Wei et al. (2011) and Cunha (2016), the blockade of A2A receptor-mediated effects appears to have little impact on synaptic transmission under baseline conditions but pharmacological or genetic block of A2A signaling attenuates synaptic plasticity/LTP at specific excitatory synapses. Neuronal and glial expression of A2A receptors can also change dramatically in response to specific conditions/stimuli such as epilepsy, further suggesting an important role in brain plasticity (Cunha, 2005).

Within the brainstem and preBötC, A1 receptor mechanisms appear to dominate but at higher levels of the CNS, possibly at the thalamus (Koos et al., 1998, 2000), A2A receptors also modulate breathing and contribute to the hypoxic respiratory depression. Injection of A2A receptor antagonists into the cisterna magna of sheep, pigs and rats reduces the hypoxic respiratory depression (Wilson et al., 2004; Koos et al., 2005; Mayer et al., 2006). Importantly, in pigs and rats the actions of the A2A antagonist on the hypoxic respiratory depression are reversed by the GABA receptor antagonist, bicuculline (Wilson et al., 2004; Mayer et al., 2006), suggesting that the A2A receptor-mediated inhibition is indirect via excitation of GABAergic neurons. The location of GABAergic neurons that receive the A2A receptor-mediated excitation is not known but it is likely rostral to the medulla and pons since A2 receptor antagonists have no effect on the actions of ADO in the rhythmic medullary slice (Mironov et al., 1999) or brainstem spinal cord preparation (Brockhaus and Ballanyi, 2000). Lack of an A2A receptor effect under these conditions does not mean A2A receptors have no role in modulation of respiratory networks. Just as A2A receptors appear important in plasticity in cortical circuits (Gomes et al., 2011; Cunha, 2016), activation of A2A receptors on phrenic motoneurons that drive the main inspiratory pump muscle of mammals can induce phrenic motor facilitation (Golder et al., 2008), a form of inducible plasticity in which inspiratory inputs to the phrenic motoneurons are potentiated. The critical role of the brainstem respiratory network in homeostasis has fueled the long-held view of the respiratory network as a hard-wired, immutable structure. This construct is gradually being replaced with the view that plasticity is likely key to the network’s ability to adapt throughout life to diverse demands that are constantly changing on multiple time scales (e.g., during development, disease, aging, transitions to altitude, exercise, phonation, speech). A role for A2A receptors in plasticity at the motor output level of this network is clear (Golder et al., 2008; Johnson and Mitchell, 2013; Fuller and Mitchell, 2017a, b). Exploration of mechanisms that might underlie frequency plasticity within respiratory rhythm generating networks is in its infancy (Johnson et al., 2019).

The actions of low-affinity A2B and A3 receptors on respiratory networks are not known, but both have been implicated in modulating synaptic plasticity through actions on A1 receptor signaling. A2B receptors are located at glutamatergic terminals of the Schaffer collateral-CA1 pyramidal neuron synapse where they counteract the predominant A1 receptor-mediated inhibition of synaptic transmission (Goncalves et al., 2015). Similarly, an A3 receptor antagonist was reported to reduce the A1 receptor-mediated inhibition of excitatory inputs at these same synapses (Dunwiddie et al., 1997a; Lopes et al., 2003).

In summary, multiple lines of evidence indicate that ADOe plays an important role in modulating respiratory network activity and in shaping the hypoxic ventilatory response [see Ballanyi (2004) for review of other modulators that may contribute]. ADOe, whether applied exogenously or derived from the hydrolysis of extracellular ATP or from accumulated intracellular ADO that is transported to the extracellular compartment via equilibrative nucleoside transporters, depresses ventilation in virtually all mammals tested, at fetal (Bissonnette et al., 1990, 1991; Koos and Matsuda, 1990), newborn (Hedner et al., 1984, 1985; Lagercrantz et al., 1984; Runold et al., 1986, 1989; Herlenius et al., 1997), and adult stages (Eldridge et al., 1984; Wessberg et al., 1984; Yamamoto et al., 1994). In addition, despite significant species differences in the sensitivity of the secondary hypoxic respiratory depression to ADO antagonists (reviewed in Ballanyi, 2004), ADO receptor antagonists consistently attenuate the secondary hypoxic respiratory depression (Runold et al., 1989; Bissonnette et al., 1990, 1991; Koos and Matsuda, 1990; Yan et al., 1995; Koos and Maeda, 2001; Koos et al., 2005) and counteract respiratory depression in apnea of prematurity (Bhatt-Mehta and Schumacher, 2003). Despite this abundance of data (Koos et al., 2001, 2005; Wilson et al., 2004; Martin and Abu-Shaweesh, 2005; Funk, 2013; Burnstock and Dale, 2015), the relative contribution of ADOe and the four subtypes of ADO (P1) receptor to the respiratory depression and their site(s) of action are not clear. Variations in protocol, drugs, and developmental stage as well as species differences confound the animal literature (Funk, 2013; Heitzmann et al., 2016). A1 receptor signaling appears to dominate in the preBötC (Lorier et al., 2007, 2008; Huxtable et al., 2009; Zwicker et al., 2011; Angelova et al., 2015), where it may be the only P1 receptor mechanism operating, at least in newborn rodents (rats and mice) (Mironov et al., 1999; Brockhaus and Ballanyi, 2000). Even within the confines of the preBötC, the action of A1 receptor activation varies developmentally and between species (Herlenius et al., 1997, 2002; Herlenius and Lagercrantz, 1999; Huxtable et al., 2009; Zwicker et al., 2011). There is significant need to understand in many species, throughout development, how the effects of ADO on preBötC activity change, as well as the magnitude of the increase in ventilation that can be achieved by inhibiting ADO receptors within the preBötC. Outside the preBötC, the neuroglial substrate responsible for the A2A receptor-mediated component of the hypoxic respiratory depression has yet to be defined and nothing is known in respiratory networks of the potential actions of A2B and A3 receptors.

Despite the efficacy of ADO receptor antagonists, especially caffeine, as respiratory stimulants that are highly effective in apnea of prematurity, ADO receptors may not be the best target for long-term manipulation of the balance between ATP and ADO signaling. First, as mentioned above, the observation that not all apnea of prematurity patients respond to ADO receptor antagonists highlights the need for alternate, non-ADO receptor focused methods of manipulating this balance. Second, P1 receptors are expressed throughout the body and brain, and potential for cardiovascular side-effects is high (Stella et al., 1993). Thus, even if more selective, higher affinity A1 receptor antagonists/agonists become available, it is unlikely that their oral or intravenous delivery would alter breathing without significant side-effects. A2A receptors are less widely expressed than A1 receptors but manipulation of their activity as a means to counteract hypoxic respiratory depression is not known. Blockade of presynaptic A2A receptor actions (which will inhibit glutamatergic transmission) is generally considered as neuroprotective but activation, rather than inhibition of post- and extrasynaptic A2A receptors may be beneficial (Gomes et al., 2011). In the context of Huntington’s Disease, it remains unclear whether A2A agonism or antagonism is clinically favorable. In contrast, A2A receptor antagonists improve motor performance in animal models of Parkinson’s Disease (Gomes et al., 2011), and are under examination for their potential clinical utility as a therapeutic strategy for Parkinson’s Disease (Takahashi et al., 2018). In the context of SUDEP, caffeine increases survival time in animal models, hypothetically by reducing the inhibitory actions of ADOe on breathing (Shen et al., 2010). However, caffeine (Chroscinska-Krawczyk et al., 2011), or treatments that reduce ADOe, promote seizure (Fedele et al., 2005).

#### Sources of Extracellular Adenosine

A key piece of information lacking for the preBötC (and all parts of the respiratory network) that is critical for the rational development of alternate approaches to manipulate the purinome to enhance P2 and inhibit P1 signaling is the source, or sources, of ADOe. The source of endogenous ADOe during normal synaptic activity, during hypoxia or during any physiological/pathophysiological process is not known. Clearly the source of elevated endogenous ADOe (e.g., via export of ADOi or degradation of ATPe) will dictate the strategies that can be used to modify ADOe. ADOe can derive from multiple sources and release mechanisms that may differ depending on the nature and severity/duration of the stimulus (synaptic activity, hypoxia/ischemia, inflammation, excitotoxicity/traumatic brain injury), brain region and even cell type (Latini and Pedata, 2001). That the cellular source of ADOe can vary with stimulus was elegantly demonstrated when cultured neurons, astrocytes, and microglia (from rat) were deprived of oxygen-glucose (OGD, to model energy failure), exposed to H_2_O_2_ (to simulate oxidative stress), or given glutamate (to induce excitotoxicity) (Jackson et al., 2017). Cultured neurons were the primarily source of ADOe during OGD or glutamate exposure while microglia were the main source during oxidative stress. As reviewed by Latini and Pedata (2001) and in agreement with early studies in culture and using hippocampal slices (Meghji et al., 1989; Lloyd et al., 1993; Brundege and Dunwiddie, 1996), a majority of studies indicate that during ischemic or hypoxic conditions the main source of ADOe is formed intracellularly and exported. Importantly, while glia may be a dominant source of ADOe during hypoxia, elevated ADOe during hypoxia could come from endothelial cells, glial cells, and also neurons from anywhere along their cell membrane; i.e., not limited to synapses (Latini and Pedata, 2001). ADO release during hypoxia is only partially sensitive to TTX and Ca^2+^-independent. This pattern of release differs markedly from normoxic conditions when depolarizing stimuli evoke an ADOe increase that is completely blocked by TTX, Ca^2+^-sensitive and derived from presynaptic neuronal release of ATP that is subsequently degraded (i.e., ADOe production is sensitive to ecto-5-nucleotidase inhibitors) (Meghji et al., 1989; Lloyd et al., 1993; Brundege and Dunwiddie, 1996; Latini and Pedata, 2001; Parkinson et al., 2005; Wall and Dale, 2008; Zhang et al., 2012; Lietsche et al., 2016). The proposal that ADO can be released via exocytosis from synaptic vesicles (Wall and Dale, 2007, 2008; Klyuch et al., 2011, 2012; Corti et al., 2013) remains controversial, reflecting in part the speed with which ATP can be converted into ADOe and therefore the difficulty of excluding ATP as a source of ADOe (Cunha, 2016). It was shown early on that the actions of ATP at P2 receptors in different brain regions are followed by inhibitory actions that are caused by the rapid degradation of ATP via ectonucleotidases into ADO and activation of P1 receptors (Dunwiddie et al., 1997b; Cunha et al., 1998). The kinetics of this biphasic ATP-ADOe signaling action appear strongly influenced by the properties of ecto-5′-nucleotidase, the main extracellular enzyme that converts AMP into ADO. During periods of intense presynaptic activity and high ATP/ADP levels, it was proposed that ADO production from AMP is delayed until activity ceases and ATP/ADP levels decline, which removes the ATP/ADP inhibition of ecto-5′-nucleotidase (Dunwiddie et al., 1997b; Latini and Pedata, 2001). Such a biphasic response is clearly seen when ATP is applied exogenously into the preBötC of neonatal rats, but not in Swiss CD mice where the excitatory actions of ATP and inhibitory actions of ADOe more or less cancel each other out (Zwicker et al., 2011). This difference may reflect differential expression of ectoATPases in the two species. As mentioned above, the dominant ectonucleotidase transcript in mice is TNAP, which converts ATPe directly to ADOe. The dominant isoform in rat is ENTPDase 2, which preferentially produces ADP from ATP (Zwicker et al., 2011). The kinetics of the ATP/ADO interaction that evolves in the preBötC in response to endogenously released ATP, and the involvement of specific ectoATPases in this relationship is not known. However, there is evidence that enzyme activity may be distributed to achieve spatially specific adenosine receptor activation. In hippocampus, production of ADOe by ecto-5′-nucleotidase has presynaptic inhibitory actions but also contributes to A2A receptor-mediated long-term potentiation of NMDA EPSCs (Rebola et al., 2008). In the striatum, ecto-5′-nucleotidase is localized to astrocytes but also prominently localized to A2A receptor-containing postsynaptic terminals where it mediates hypolocomotion and modulates working memory (Augusto et al., 2013).

Although the majority of studies (primarily in hippocampus) indicate that the main source of ADOe during ischemic or hypoxic conditions is formed intracellularly (Meghji et al., 1989; Lloyd et al., 1993; Brundege and Dunwiddie, 1996), this may not be case for regions involved in respiratory control. During hypoxia in fetal sheep, an important source of ADOe in the thalamic parafascicular locus, where A2A receptors contribute to the secondary hypoxic respiratory depression (Koos et al., 1998), appears to be extracellular; i.e., the hypoxia-induced increase in ADOe is unaffected by ENT inhibitors but reduced by inhibitors of ecto-5′- nucleotidase (Koos et al., 1997). Within the preBötC we also propose an extracellular source of ADOe during hypoxia; i.e., that hypoxia evokes ATP release from astrocytes via exocytosis, which is then degraded to ADOe. Whether ATPe released via exocytosis, and the subsequently produced ADOe, will diffuse to extrasynaptic receptors and have broad, generalized effects over the entire network, or have localized actions on specific preBötC neurons/synapses is not known. However, within the framework of the localized, bidirectional communication that is proposed to occur between pre- and postsynaptic terminals and astrocytes in the tripartite synapse (Araque et al., 1999; Covelo and Araque, 2018), and the observation that preBötC respiratory neurons differ markedly in their sensitivity to ATP/P2Y_1_ and ADO receptor activation (Herlenius and Lagercrantz, 1999; Mironov et al., 1999; Thomas and Spyer, 2000; Lorier et al., 2007, 2008), neuroglial interactions in the preBötC are likely to be highly specialized, regionally and functionally; i.e., the effects of ATP/ADP/ADO released from astrocytes during hypoxia are likely to reflect activation of specific synapses and neurons.

### Equilibrative Nucleoside Transporters

There are four ENT isoforms that move ADO passively, and bidirectionally across cell membranes. The direction of ADO transport is determined by the ADO concentration gradient. ENT1 and 2 carry out the majority of ADO transport across the outer cell membrane (Parkinson et al., 2011). ENT3 is intracellular and irrelevant in determining ADOe while ENT4 appears to function primarily in monoamine transport. Three isoforms of concentrative nucleoside transporters (CNTs) in the CNS are Na^+^-coupled and indirectly consume ATP to move ADO against its concentration gradient, but there is no evidence that CNTs regulate ADOe under normal physiological conditions (Parkinson et al., 2011; Chu et al., 2013). Thus, our focus is on ENTs. They play a key role in maintaining ADOe under baseline conditions, as demonstrated by > 4-fold increases in ADOe in the striatum of freely behaving rats following transporter inhibition (Ballarin et al., 1991). Thus, they are increasingly recognized as potential targets in drug discovery, in part because ADOe is implicated in a range of significant health issues, including cardiovascular disease and sleep disorders (King et al., 2006).

Manipulation of ENTs to control ADOe requires that the source of the increased ADOe during hypoxia is known (Pearson et al., 2003; Pascual et al., 2005; Martin et al., 2007; Funk, 2013). As discussed above (*Sources of extracellular ADO*), if the main source of ADOe is from the ENT-mediated export of ADOi that accumulates during hypoxia, then ENT inhibition would be required to reduce ADOe in the preBötC. If, however, the main source of ADOe is from degradation of ATPe, then the objective would be to potentiate inward ENT-mediated transport and facilitate the clearance of ADOe. Multiple sources and mechanisms can contribute to an hypoxia-induced increase in ADOe. While ENT-mediated export may be a dominant source of ADOe during hypoxia/ischemia in some parts of the brain (Meghji et al., 1989; Lloyd et al., 1993; Brundege and Dunwiddie, 1996; Latini and Pedata, 2001), this may not be the case in the respiratory network. The role of ENTs in regulating ADOe during hypoxia has not been examined specifically in the preBötC. Under baseline conditions in the brainstem spinal cord of neonatal rats, the ENT inhibitor, dipyridamole, decreases inspiratory-related burst frequency, indicating that at least under these conditions ENT activity is important for clearing ADOe (Herlenius et al., 1997). In fetal sheep during hypoxia, however, a major source of ADOe implicated in the hypoxic respiratory depression (Koos et al., 1998) is from degradation of extracellular adenine nucleotides (Koos et al., 1997).

**FIGURE 4:**
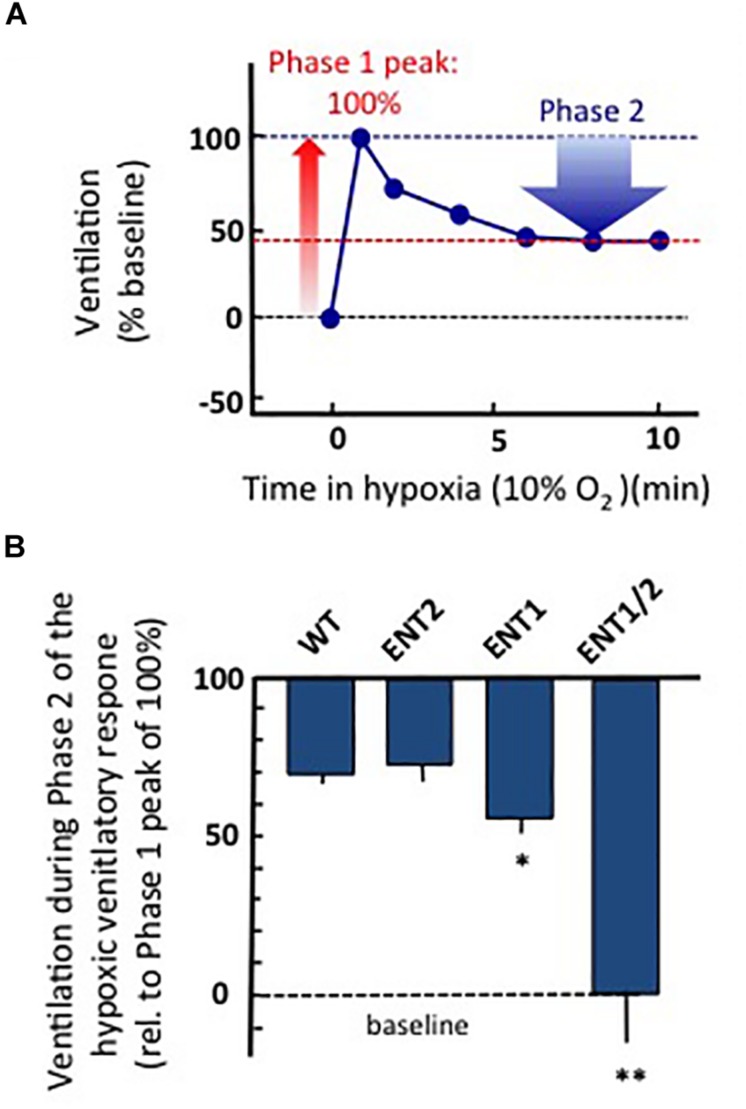
Global knock out of ENTs increases the secondary hypoxic respiratory depression. **(A)** Cartoon showing the biphasic hypoxic ventilatory response, which comprises a Phase 1 increase followed by a secondary depression to a lower, steady-state level of ventilation in Phase 2. The Phase 1 increase was reported as 100%. Ventilation during Phase 2 was calculated as a percentage of the peak increase during Phase 1. In this mock example, ventilation during phase 2 was ∼45% of the phase 1 increase; i.e., ventilation fell by ∼55% from the peak during the hypoxic respiratory depression. **(B)** The level of ventilation (measured using whole-body plethysmography) during Phase 2 of the hypoxic ventilatory response is reported relative to the Phase 1 peak increase for unanesthetized wild type (WT), ENT2, ENT1, and ENT1/2 double knockout mice. ^∗^Represents sig. difference from WT, *p* < 0.05 or ^∗∗^*p* < 0.01. ANOVA was used in conjunction with Bonferroni *post hoc* multiple comparison test.

To gain insight into the potential source of ADOe during hypoxia in rodents, we have recently used whole-body plethysmography (Zwicker et al., 2014; Angelova et al., 2015) to compare the hypoxic ventilatory responses of adult, wild-type and three strains of transgenic mice from which ENT1, ENT2 or ENT1 and 2 were both knocked out from birth. All experiments were carried out in accordance with the guidelines of the Canadian Council on Animal Care and were approved by the University of Alberta Animal Ethics Committee (Protocols AUP256). Animals were placed in a whole-body, plexiglass plethysmograph that had inflow and outflow ports for continuous delivery of a steady flow of air (21% O_2_, balance N_2_ or 10% O_2_/balance N_2_). Pressure changes in the chamber were recorded with a pressure transducer (DP 103; Validyne, Northridge, CA), signal conditioner (CD-15; Validyne), analog-digital board (Digidata 1322), and Axoscope software (Molecular Devices, Sunnyvale, CA). Animals were allowed 30 min to become accustomed to the chamber. Baseline ventilatory parameters (frequency, relative tidal volume and relative minute ventilation) were then recorded for 10 min and gas composition was changed to 10% O_2_/balance N_2_ for 10 min and then returned to 21% O_2_. Air flow and composition were controlled to produce a rapid transition from 21 to 10% O_2_ (< 15 s). Note that measurements of frequency are quantitative while changes in tidal volume and minute ventilation (the product of frequency and tidal volume) are semiquantitative (reported relative to baseline). The increase in ventilation from baseline to the peak observed during Phase 1 of the hypoxic ventilatory response was reported as 100%. Ventilation during Phase 2 (average between min 6–10 of hypoxic exposure) was calculated as a percentage of the peak increase recording during Phase 1 (Figure 4A). All strains showed a biphasic ventilatory response to hypoxia but the magnitude of the hypoxic respiratory depression was greatest in the ENT1/2 KO mice > ENT1 KO > ENT2 KO ≈ wild type mice (Figure 4B). The size of the blue bar indicates the magnitude of the depression; i.e., ventilation of the WT mice during Phase 2 was ∼70% of the peak seen during Phase 1 (i.e., during the hypoxic respiratory depression, ventilation fell ∼ 30% from the peak). In contrast, ventilation in the ENT1/2 KOs was 0% of peak, indicating that during Phase 2 ventilation fell all the way to baseline levels (100% depression). These data suggest that the main source of ADOe causing the hypoxic depression comes from breakdown of ATPe. If ADOe came via ENT-mediated outward transport of ADOi, one would expect the opposite – that ADOe would not increase in the ENT KOs and there would be no depression. The main limitation with these data is that ENTs are knocked out globally from birth in these animals, including from the carotid body. Thus, additional work with conditional and/or localized deletion/inhibition of ENT activity will be required to fully evaluate in respiratory networks the source of elevated ADOe during hypoxia, and the role of ENTs in the hypoxic ventilatory response. Such information is required to inform strategies on how to manipulate ENT function to reduce hypoxic respiratory depression.

### Adenosine Kinase

The impact of manipulating ENT activity on ADOe emphasizes the importance of ADOe metabolic clearance mechanisms in regulating ADOe under baseline, hypoxic and pathological conditions. ENT activity, however, is only part of the ADOe clearance equation. The direction of ADO transport by ENTs is determined by the ADO concentration gradient across the cellular membrane; ADOi must be maintained below ADOe for ENTs to effectively remove ADOe. As demonstrated in rat hippocampal slices (Lloyd and Fredholm, 1995), ADOi and ADOe are influenced by several intracellular enzymes. These include S-adenosyl homocysteine hydrolase, the low affinity, high capacity metabolic enzyme adenosine deaminase, that converts ADO into inosine, and the high affinity, low capacity enzyme ADK that phosphorylates ADOi to AMPi. Our discussion focuses on ADK because it has a higher affinity for ADO than adenosine deaminase, and in hippocampal and cortical networks it is the most important enzyme affecting intracellular levels of ADO and in controlling neuronal excitability and ADO signaling (Pak et al., 1994; Boison, 2013b). It is important to note that in the adult brain ADK is primarily expressed in astrocytes. Therefore ADOe and hence activation of ADO receptors on neurons is under the control of astrocyte-based metabolic clearance of ADOe. Dysregulation of ADK may be causative in some forms of epilepsy. The ADK hypothesis of epilepsy posits that acute insults to the brain lead to an initial and transient downregulation of ADK and an acute elevation of ADOe that is protective in the short term. However, the acute changes trigger maladaptive long term changes of the purinome including downregulation of astrocytic A1 receptors, upregulation of A2A receptors, and astrogliosis in conjunction with chronic overexpression of ADK, reduced ADOe and ultimately spontaneous focal seizure activity in astrogliotic regions (Boison, 2008). Pharmacological inhibition of ADK (Kowaluk and Jarvis, 2000), or ADO augmentation therapies developed to inhibit ADK and raise ADOe greatly reduce seizures in animal models of epilepsy (Boison, 2012b), whereas transgenic overexpression of ADK triggers seizures (Fedele et al., 2005). Whether ADK inhibition increases the risk of respiratory depression, and therefore SUDEP, is not known.

In fact, virtually nothing is known about the impact of ADK on either the baseline control of breathing or the hypoxic ventilatory response. Most exciting in terms of respiratory control is that the ADK system changes dramatically during postnatal development (Boison, 2013b). ADK comes in long nuclear and short cytoplasmic isoforms, ADK-L and ADK-S, respectively. The nuclear isoform ADK-L has an epigenetic role as regulator of DNA methylation, whereas the cytoplasmic form ADK-S is responsible for regulating extracellular levels of adenosine and ADO receptor activation (Boison, 2013b; Williams-Karnesky et al., 2013). During fetal and early postnatal brain development there is a shift in the expression from ADK-L to more ADK-S and from neuronal expression toward astrocytic expression, suggesting a functional change from an epigenetic regulator of brain development toward a regulator of extracellular adenosine. Therefore, the major system responsible for clearing ADOe in the hippocampus is immature at birth. If the maturation of ADK follows a similar developmental profile in the brainstem respiratory network, it could contribute to the greater susceptibility of premature animals to hypoxic respiratory depression (Moss and Inman, 1989). Tools developed to study the role of ADK in epilepsy, including transgenic mouse lines (Tronche et al., 1999; Boison et al., 2002; Fedele et al., 2004) and adenoassociated viral vectors that knockdown ADK specifically in neurons or astrocytes (Theofilas et al., 2011), should be very instructive in advancing our understanding of the role played by ADK in respiratory control and the hypoxic ventilatory response.

In summary, we have reviewed evidence that purinergic signaling within the preBötC network modulates breathing rhythm and shapes the ventilatory response to hypoxia. Purinergic signaling in other brain regions may contribute to this reflex, but our discussion has focused on the preBötC because it is within this region that we have the strongest evidence that P2 and P1 receptor signaling and ectonucleotidase activity shape the hypoxic ventilatory response. Even for these components of the purinome many questions remain. These include: (i) the identity of signaling cascades and ion channels through which the different receptors modulate breathing rhythm; (ii) the significance of ectonucleotidase diversity for respiratory control; and (iii) the source of ADOe during hypoxia – degradation of ATPe or outward ENT-mediated transport of ADOi. Studies exploring the roles in the hypoxic ventilatory response of ADO transporters (the ENTs), and intracellular enzymes important in controlling ADOi and ADOe clearance (e.g., ADK) are only in their infancy. The developmental dynamics of purinergic signaling in the respiratory network also requires investigation, especially given the potential involvement of the purinome in the greater susceptibility of premature and newborn mammals to hypoxic respiratory depression. Additional key areas of future investigation not discussed above include how the effects of purinergic signaling on cerebral vasculature will affect network excitability, and how the mechanisms and dynamics of purinergic signaling differ between conditions like hypoxia, when ATP is released through physiologically relevant processes, and traumatic injury where ATP is released in high concentration from damaged/ruptured cells including red blood cells? A key translational challenge is to selectively manipulate the balance between ATP/ADO signaling to stimulate ventilation without simultaneously interfering with the beneficial actions of ADOe in other brain regions. To do so will require detailed understanding, not only of the purinome within the preBötC and other respiratory nuclei, but the rest of the brain as well.

## Ethics Statement

All experiments were carried out in accordance with the guidelines of the Canadian Council on Animal Care and were approved by the University of Alberta Animal Ethics Committee (Protocols AUP256).

## Author Contributions

GF wrote the first draft of the manuscript. RR, TA, and GF contributed to the conception and design of the experiments described in Figure 3, while RR and TA completed these studies and performed the statistical analysis. RR, YZ, AM, VB, AK, SF, MH, and AT wrote the sections of the manuscript. JY and CC contributed the ENT knockout mice. DB consulted on ADK biochemistry and implications for respiratory control, and edited the manuscript. All authors contributed to the manuscript conception and revision, and read and approved the submitted version.

## Conflict of Interest Statement

DB is a co-founder of PrevEp LLC, and serves as scientific advisor for Hoffmann LaRoche. The remaining authors declare that the research was conducted in the absence of any commercial or financial relationships that could be construed as a potential conflict of interest.

## References

[B1] Abbracchio M. P. Brambilla R. Ceruti S. Kim H. O. von Lubitz D. K. Jacobson K. A. (1995). G protein-dependent activation of phospholipase C by adenosine A3 receptors in rat brain. *Mol. Pharmacol.* 48 1038–1045. 8848003

[B2] Abbracchio M. P. Burnstock G. Boeynaems J. M. Barnard E. A. Boyer J. L. Kennedy C. (2006). International union of pharmacology LVIII: update on the P2Y G protein-coupled nucleotide receptors: from molecular mechanisms and pathophysiology to therapy. *Pharmacol. Rev.* 58 281–341. 10.1124/pr.58.3.3 16968944 PMC3471216

[B3] Abbracchio M. P. Burnstock G. Verkhratsky A. Zimmermann H. (2009). Purinergic signalling in the nervous system: an overview. *Trends Neurosci.* 32 19–29. 10.1016/j.tins.2008.10.001 19008000

[B4] al-Rashida M. Iqbal J. (2014). Therapeutic potentials of ecto-nucleoside triphosphate diphosphohydrolase, ecto-nucleotide pyrophosphatase/phosphodiesterase, ecto-5’-nucleotidase, and alkaline phosphatase inhibitors. *Med. Res. Rev.* 34 703–743. 10.1002/med.21302 24115166

[B5] Ambrosio A. F. Malva J. O. Carvalho A. P. Carvalho C. M. (1997). Inhibition of N-,P/Q- and other types of Ca2+ channels in rat hippocampal nerve terminals by the adenosine A1 receptor. *Eur. J. Pharmacol.* 340 301–310. 10.1016/s0014-2999(97)01451-9 9537827

[B6] Angelova P. R. Kasymov V. Christie I. Sheikhbahaei S. Turovsky E. Marina N. (2015). Functional oxygen sensitivity of astrocytes. *J. Neurosci.* 35 10460–10473. 10.1523/JNEUROSCI.0045-15.2015 26203141 PMC4510287

[B7] Aoyama T. Koga S. Nakatsuka T. Fujita T. Goto M. Kumamoto E. (2010). Excitation of rat spinal ventral horn neurons by purinergic P2X and P2Y receptor activation. *Brain Res.* 1340 10–17. 10.1016/j.brainres.2010.04.053 20423703

[B8] Araque A. Parpura V. Sanzgiri R. P. Haydon P. G. (1999). Tripartite synapses: glia, the unacknowledged partner. *Trends Neurosci.* 22 208–215. 10.1016/s0166-2236(98)01349-6 10322493

[B9] Augusto E. Matos M. Sevigny J. El-Tayeb A. Bynoe M. S. Muller C. E. (2013). Ecto-5’-nucleotidase (CD73)-mediated formation of adenosine is critical for the striatal adenosine A2A receptor functions. *J. Neurosci.* 33 11390–11399. 10.1523/JNEUROSCI.5817-12.2013 23843511 PMC3724543

[B10] Baertsch N. A. Baertsch H. C. Ramirez J. M. (2018). The interdependence of excitation and inhibition for the control of dynamic breathing rhythms. *Nat. Commun.* 9:843. 10.1038/s41467-018-03223-x 29483589 PMC5827754

[B11] Ballanyi K. (2004). Neuromodulation of the perinatal respiratory network. *Curr. Neuropharmacol.* 2 221–243. 10.2174/1570159043476828 30509012

[B12] Ballarin M. Fredholm B. B. Ambrosio S. Mahy N. (1991). Extracellular levels of adenosine and its metabolites in the striatum of awake rats: inhibition of uptake and metabolism. *Acta Physiol. Scand.* 142 97–103. 10.1111/j.1748-1716.1991.tb09133.x 1877368

[B13] Bellingham M. C. Berger A. J. (1994). Adenosine suppresses excitatory glutamatergic inputs to rat hypoglossal motoneurons in vitro. *Neurosci. Lett.* 177 143–146. 10.1016/0304-3940(94)90065-5 7824167

[B14] Bhatt-Mehta V. Schumacher R. E. (2003). Treatment of apnea of prematurity. *Paediatr. Drugs* 5 195–210. 10.2165/00148581-200305030-00006 12608884

[B15] Bissonnette J. M. (2000). Mechanisms regulating hypoxic respiratory depression during fetal and postnatal life. *Am. J. Physiol. Regul. Integr. Comp. Physiol.* 278 R1391–R1400. 10848503 10.1152/ajpregu.2000.278.6.R1391

[B16] Bissonnette J. M. Hohimer A. R. Knopp S. J. (1991). The effect of centrally administered adenosine on fetal breathing movements. *Respir. Physiol.* 84 273–285. 10.1016/0034-5687(91)90123-z 1876764

[B17] Bissonnette J. M. Hohimer R. Chao C. R. Knopp S. J. Notoroberto N. F. (1990). Theophylline stimulates fetal breathing movements during hypoxia. *Paediatr. Res.* 28 83–86. 10.1203/00006450-199028020-00002 2395607

[B18] Boison D. (2008). The adenosine kinase hypothesis of epileptogenesis. *Prog. Neurobiol.* 84 249–262. 10.1016/j.pneurobio.2007.12.002 18249058 PMC2278041

[B19] Boison D. (2011). Methylxanthines, seizures, and excitotoxicity. *Handb. Exp. Pharmacol.* 2011 251–266. 10.1007/978-3-642-13443-2-9 20859799 PMC2945384

[B20] Boison D. (2012a). A Breather for SUDEP. *Epilepsy. Curr.* 12 111–112. 10.5698/1535-7511-12.3.111 22688854 PMC3367419

[B21] Boison D. (2012b). “Adenosine augmentation therapy,” in *Jasper’s Basic Mechanisms of the Epilepsies*, 4th Edn, eds Noebels J. L. Avoli M. Rogawski M. A. Olsen R. W. Delgado-Escueta A. V. (Oxford: OUP).

[B22] Boison D. (2013a). Adenosine and seizure termination: endogenous mechanisms. *Epilepsy. Curr.* 13 35–37. 10.5698/1535-7511-13.1.35 23447738 PMC3577085

[B23] Boison D. (2013b). Adenosine kinase: exploitation for therapeutic gain. *Pharmacol. Rev.* 65 906–943. 10.1124/pr.112.006361 23592612 PMC3698936

[B24] Boison D. (2016). Adenosinergic signaling in epilepsy. *Neuropharmacology* 104 131–139. 10.1016/j.neuropharm.2015.08.046 26341819 PMC4775444

[B25] Boison D. Scheurer L. Zumsteg V. Rulicke T. Litynski P. Fowler B. (2002). Neonatal hepatic steatosis by disruption of the adenosine kinase gene. *Proc. Natl. Acad. Sci. U.S.A.* 99 6985–6990. 10.1073/pnas.092642899 11997462 PMC124515

[B26] Brockhaus J. Ballanyi K. (2000). Anticonvulsant A(1) receptor-mediated adenosine action on neuronal networks in the brainstem-spinal cord of newborn rats. *Neuroscience* 96 359–371. 10.1016/s0306-4522(99)00544-8 10683576

[B27] Brundege J. M. Dunwiddie T. V. (1996). Modulation of excitatory synaptic transmission by adenosine released from single hippocampal pyramidal neurons. *J. Neurosci.* 16 5603–5612. 10.1523/jneurosci.16-18-05603.1996 8795616 PMC6578976

[B28] Burnstock G. (2006). Purinergic P2 receptors as targets for novel analgesics. *Pharmacol. Ther.* 110 433–454. 10.1016/j.pharmthera.2005.08.013 16226312

[B29] Burnstock G. (2015). Purinergic signalling and the autonomic nervous system in health and disease. *Auton. Neurosci.* 191:1. 10.1016/j.autneu.2015.05.006 26026395

[B30] Burnstock G. Dale N. (2015). Purinergic signalling during development and ageing. *Purinergic. Signal.* 11 277–305. 10.1007/s11302-015-9452-9459 25989750 PMC4529855

[B31] Burnstock G. Fredholm B. B. Verkhratsky A. (2011). Adenosine and ATP receptors in the brain. *Curr. Top. Med. Chem.* 11 973–1011. 10.2174/156802611795347627 21401499

[B32] Canas P. M. Porciuncula L. O. Simoes A. P. Augusto E. Silva H. B. Machado N. J. (2018). Neuronal adenosine A2A receptors are critical mediators of neurodegeneration triggered by convulsions. *eNeuro* 5:ENEURO.0385-18.2018. 10.1523/ENEURO.0385-18.2018 30627646 PMC6325550

[B33] Chroscinska-Krawczyk M. Jargiello-Baszak M. Walek M. Tylus B. Czuczwar S. J. (2011). Caffeine and the anticonvulsant potency of antiepileptic drugs: experimental and clinical data. *Pharmacol. Rep.* 63 12–18. 10.1016/s1734-1140(11)70394-2 21441607

[B34] Chu S. Xiong W. Zhang D. Soylu H. Sun C. Albensi B. C. (2013). Regulation of adenosine levels during cerebral ischemia. *Acta Pharmacol. Sin.* 34 60–66. 10.1038/aps.2012.127 23064722 PMC4086501

[B35] Comer A. M. Perry C. M. Figgitt D. P. (2001). Caffeine citrate: a review of its use in apnoea of prematurity. *Paediatr. Drugs* 3 61–79. 10.2165/00128072-200103010-00005 11220405

[B36] Conde S. V. Monteiro E. C. Sacramento J. F. (2017). Purines and carotid body: new roles in pathological conditions. *Front. Pharmacol.* 8:913. 10.3389/fphar.2017.00913 29311923 PMC5733106

[B37] Corti F. Cellai L. Melani A. Donati C. Bruni P. Pedata F. (2013). Adenosine is present in rat brain synaptic vesicles. *Neuroreport* 24 982–987. 10.1097/WNR.0000000000000033 24051680

[B38] Covelo A. Araque A. (2018). Neuronal activity determines distinct gliotransmitter release from a single astrocyte. *Elife* 7 e32237. 10.7554/eLife.32237 29380725 PMC5790377

[B39] Cunha R. A. (2005). Neuroprotection by adenosine in the brain: from A(1) receptor activation to A (2A) receptor blockade. *Purinergic Signal.* 1 111–134. 10.1007/s11302-005-0649-641 18404497 PMC2096528

[B40] Cunha R. A. (2016). How does adenosine control neuronal dysfunction and neurodegeneration? *J. Neurochem.* 139 1019–1055. 10.1111/jnc.13724 27365148

[B41] Cunha R. A. Sebasti A. M. Ribeiro J. A. (1998). Inhibition by ATP of hippocampal synaptic transmission requires localized extracellular catabolism by ecto-nucleotidases into adenosine and channeling to adenosine A1 receptors. *J. Neurosci.* 18 1987–1995. 10.1523/jneurosci.18-06-01987.1998 9482785 PMC6792930

[B42] Curran A. K. Rodman J. R. Eastwood P. R. Henderson K. S. Dempsey J. A. Smith C. A. (2000). Ventilatory responses to specific CNS hypoxia in sleeping dogs. *J. Appl. Physiol.* 88 1840–1852. 10.1152/jappl.2000.88.5.1840 10797149

[B43] Dale N. Frenguelli B. G. (2009). Release of adenosine and ATP during ischemia and epilepsy. *Curr. Neuropharmacol.* 7 160–179. 10.2174/157015909789152146 20190959 PMC2769001

[B44] Daristotle L. Engwall M. J. Niu W. Z. Bisgard G. E. (1991). Ventilatory effects and interactions with change in PaO2 in awake goats. *J. Appl. Physiol.* 71 1254–1260. 10.1152/jappl.1991.71.4.1254 1757347

[B45] de Mendonca A. Sebastiao A. M. Ribeiro J. A. (1995). Inhibition of NMDA receptor-mediated currents in isolated rat hippocampal neurones by adenosine A1 receptor activation. *Neuroreport* 6 1097–1100. 10.1097/00001756-199505300-00006 7662885

[B46] Del Negro C. A. Funk G. D. Feldman J. L. (2018). Breathing matters. *Nat. Rev. Neurosci.* 19 351–367. 10.1038/s41583-018-0003-6 29740175 PMC6636643

[B47] Dempsey J. A. Smith C. A. (2019). Update on chemoreception: influence on cardiorespiratory regulation and pathophysiology. *Clin. Chest. Med.* 40 269–283. 10.1016/j.ccm.2019.02.001 31078209 PMC6512837

[B48] Dempsey J. A. Smith C. A. Blain G. M. Xia A. Gong Y. Teodorescu M. (2012). “Role of central/peripheral chemoreceptors and their interdependence in the pathophysiology of sleep apnea,” in *Arterial Chemoreception: from Molecules to Systems*, 758 Edn, eds Nurse C. A. Gonzales C. Peers C. Prabhakar N. R. (New York, NY: Springer), 343–350.

[B49] Dempsey J. A. Veasey S. C. Morgan B. J. O’Donnell C. P. (2010). Pathophysiology of sleep apnea. *Physiol. Rev.* 90 47–112. 10.1152/physrev.00043.2008 20086074 PMC3970937

[B50] Devinsky O. (2011). Sudden, unexpected death in epilepsy. *N. Engl. J. Med.* 365 1801–1811. 10.1056/NEJMra1010481 22070477

[B51] Dixon A. K. Gubitz A. K. Sirinathsinghji D. J. Richardson P. J. Freeman T. C. (1996). Tissue distribution of adenosine receptor mRNAs in the rat. *Br. J. Pharmacol.* 118 1461–1468. 10.1111/j.1476-5381.1996.tb15561.x 8832073 PMC1909676

[B52] Dong X. W. Feldman J. L. (1995). Modulation of inspiratory drive to phrenic motoneurons by presynaptic adenosine A1 receptors. *J. Neurosci.* 15(5 Pt 1), 3458–3467. 10.1523/jneurosci.15-05-03458.1995 7538560 PMC6578246

[B53] Dunwiddie T. V. Diao L. Kim H. O. Jiang J. L. Jacobson K. A. (1997a). Activation of hippocampal adenosine A3 receptors produces a desensitization of A1 receptor-mediated responses in rat hippocampus. *J. Neurosci.* 17 607–614. 10.1523/jneurosci.17-02-00607.1997 8987783 PMC5470729

[B54] Dunwiddie T. V. Diao L. Proctor W. R. (1997b). Adenine nucleotides undergo rapid, quantitative conversion to adenosine in the extracellular space in rat hippocampus. *J. Neurosci.* 17 7673–7682. 10.1523/jneurosci.17-20-07673.1997 9315889 PMC6793930

[B55] Dunwiddie T. V. Masino S. A. (2001). The role and regulation of adenosine in the central nervous system. *Annu. Rev. Neurosci.* 24 31–55. 10.1146/annurev.neuro.24.1.31 11283304

[B56] El Yacoubi M. Ledent C. Parmentier M. Costentin J. Vaugeois J. M. (2009). Adenosine A2A receptor deficient mice are partially resistant to limbic seizures. *Naunyn Schmiedebergs Arch. Pharmacol.* 380 223–232. 10.1007/s00210-009-0426-428 19488739

[B57] El Yacoubi M. Ledent C. Parmentier M. Daoust M. Costentin J. Vaugeois J. (2001). Absence of the adenosine A(2A) receptor or its chronic blockade decrease ethanol withdrawal-induced seizures in mice. *Neuropharmacology* 40 424–432. 10.1016/s0028-3908(00)00173-8 11166335

[B58] Eldridge F. L. Millhorn D. E. Kiley J. P. (1984). Respiratory effects of a long-acting analog of adenosine. *Brain Res.* 310 273–280. 10.1016/0006-8993(84)91096-5 6428704

[B59] Fedele D. E. Gouder N. Guttinger M. Gabernet L. Scheurer L. Rulicke T. (2005). Astrogliosis in epilepsy leads to overexpression of adenosine kinase, resulting in seizure aggravation. *Brain* 128(Pt 10), 2383–2395. 10.1093/brain/awh555 15930047

[B60] Fedele D. E. Koch P. Scheurer L. Simpson E. M. Mohler H. Brustle O. (2004). Engineering embryonic stem cell derived glia for adenosine delivery. *Neurosci. Lett.* 370 160–165. 10.1016/j.neulet.2004.08.031 15488315

[B61] Feldman J. L. Del Negro C. A. (2006). Looking for inspiration: new perspectives on respiratory rhythm. *Nat. Rev. Neurosci.* 7 232–242. 16495944 10.1038/nrn1871 PMC2819067

[B62] Fredholm B. B. Abbracchio M. P. Burnstock G. Daly J. W. Harden T. K. Jacobson K. A. (1994). Nomenclature and classification of purinoceptors. *Pharmacol. Rev.* 46 143–156. 7938164 PMC4976594

[B63] Fredholm B. B. Arslan G. Halldner L. Kull B. Schulte G. Wasserman W. (2000). Structure and function of adenosine receptors and their genes. *Naunyn Schmiedebergs Arch. Pharmacol.* 362 364–374. 11111830 10.1007/s002100000313

[B64] Fredholm B. B. IJzerman A. P. Jacobson K. A. Klotz K. N. Linden J. (2001). International union of pharmacology. XXV. Nomenclature and classification of adenosine receptors. *Pharmacol. Rev.* 53 527–552. 11734617 PMC9389454

[B65] Fuller D. D. Mitchell G. S. (2017a). Respiratory neuroplasticity - overview, significance and future directions. *Exp. Neurol.* 287(Pt 2), 144–152. 10.1016/j.expneurol.2016.05.022 27208699

[B66] Fuller D. D. Mitchell G. S. (2017b). Special Issue: respiratory Neuroplasticity. *Exp. Neurol.* 287(Pt 2), 91–92. 10.1016/j.expneurol.2016.11.004 27871363

[B67] Funk G. D. (2013). Neuromodulation: purinergic signaling in respiratory control. *Compr. Physiol.* 3 331–363. 10.1002/cphy.c120004 23720290

[B68] Funk G. D. Gourine A. V. (2018a). CrossTalk proposal: a central hypoxia sensor contributes to the excitatory hypoxic ventilatory response. *J. Physiol.* 596 2935–2938. 10.1113/JP275707 29947079 PMC6068249

[B69] Funk G. D. Gourine A. V. (2018b). Rebuttal from gregory D. Funk and Alexander V. Gourine. *J. Physiol.* 596 2943–2944. 10.1113/JP276282 29947074 PMC6068248

[B70] Funk G. D. Huxtable A. G. Lorier A. R. (2008). ATP in central respiratory control: a three-part signaling system. *Respir. Physiol. Neurobiol.* 164 131–142. 10.1016/j.resp.2008.06.004 18586120

[B71] Golder F. J. Ranganathan L. Satriotomo I. Hoffman M. Lovett-Barr M. R. Watters J. J. (2008). Spinal adenosine A2a receptor activation elicits long-lasting phrenic motor facilitation. *J. Neurosci.* 28 2033–2042. 10.1523/JNEUROSCI.3570-07.2008 18305238 PMC6671860

[B72] Gomes C. V. Kaster M. P. Tome A. R. Agostinho P. M. Cunha R. A. (2011). Adenosine receptors and brain diseases: neuroprotection and neurodegeneration. *Biochim. Biophys. Acta* 1808 1380–1399. 10.1016/j.bbamem.2010.12.001 21145878

[B73] Goncalves F. Q. Pires J. Pliassova A. Beleza R. Lemos C. Marques J. M. (2015). Adenosine A2b receptors control A1 receptor-mediated inhibition of synaptic transmission in the mouse hippocampus. *Eur. J. Neurosci.* 41 878–888. 10.1111/ejn.12851 25704806

[B74] Gourine A. V. Funk G. D. (2017). On the existence of a central respiratory oxygen sensor. *J. Appl. Physiol.* 123 1344–1349. 10.1152/japplphysiol.00194.2017 28522760 PMC5792097

[B75] Gourine A. V. Llaudet E. Dale N. Spyer K. M. (2005). Release of ATP in the ventral medulla during hypoxia in rats: role in hypoxic ventilatory response. *J. Neurosci.* 25 1211–1218. 10.1523/jneurosci.3763-04.2005 15689558 PMC6725960

[B76] Greene R. W. Haas H. L. (1991). The electrophysiology of adenosine in the mammalian central nervous system. *Prog. Neurobiol.* 36 329–341. 10.1016/0301-0082(91)90005-l 1678539

[B77] Guyenet P. G. (2006). The sympathetic control of blood pressure. *Nat. Rev. Neurosci.* 7 335–346. 10.1038/nrn1902 16760914

[B78] Haas H. L. Selbach O. (2000). Functions of neuronal adenosine receptors. *Naunyn Schmiedebergs Arch. Pharmacol.* 362 375–381. 10.1007/s002100000314 11111831

[B79] Haselkorn M. L. Shellington D. K. Jackson E. K. Vagni V. A. Janesko-Feldman K. Dubey R. K. (2010). Adenosine A1 receptor activation as a brake on the microglial response after experimental traumatic brain injury in mice. *J. Neurotrauma* 27 901–910. 10.1089/neu.2009.1075 20121416 PMC2943944

[B80] Hedner T. Hedner J. Bergman B. Mueller R. A. Jonason J. (1985). Characterization of adenosine-induced respiratory depression in the preterm rabbit. *Biol. Neonate* 47 323–332. 10.1159/000242135 3839698

[B81] Hedner T. Hedner J. Jonason J. Wessberg P. (1984). Effects of theophylline on adenosine-induced respiratory depression in the preterm rabbit. *Eur. J. Respir. Dis.* 65 153–156. 6698140

[B82] Heitzmann D. Buehler P. Schweda F. Georgieff M. Warth R. Thomas J. (2016). The in vivo respiratory phenotype of the adenosine A1 receptor knockout mouse. *Respir. Physiol. Neurobiol.* 222 16–28. 10.1016/j.resp.2015.11.005 26593641

[B83] Herlenius E. Aden U. Tang L. Q. Lagercrantz H. (2002). Perinatal respiratory control and its modulation by adenosine in neonatal rat. *Ped Res.* 51 4–12. 10.1203/00006450-200201000-00004 11756633

[B84] Herlenius E. Lagercrantz H. (1999). Adenosinergic modulation of respiratory neurones in the neonatal rat brainstem in vitro. *J. Physiol.* 518(Pt 1), 159–172. 10.1111/j.1469-7793.1999.0159r.x 10373698 PMC2269420

[B85] Herlenius E. Lagercrantz H. Yamamoto Y. (1997). Adenosine modulates inspiratory neurons and the respiratory pattern in the brainstem of neonatal rats. *Pediatr. Res.* 42 46–53. 10.1203/00006450-199707000-00008 9212036

[B86] Huxtable A. G. Zwicker J. D. Alvares T. S. Ruangkittisakul A. Fang X. Hahn L. B. (2010). Glia contribute to the purinergic modulation of inspiratory rhythm-generating networks. *J. Neurosci.* 30 3947–3958. 10.1523/JNEUROSCI.6027-09.2010 20237265 PMC6632270

[B87] Huxtable A. G. Zwicker J. D. Poon B. Y. Pagliardini S. Vrouwe S. Q. Greer J. J. (2009). Tripartite purinergic modulation of central respiratory networks during perinatal development: the influence of ATP, ectonucleotidases, and ATP metabolites. *J. Neurosci.* 29 14713–14725. 10.1523/JNEUROSCI.2660-09.2009 19940166 PMC6666021

[B88] Jackson E. K. Kotermanski S. E. Menshikova E. V. Dubey R. K. Jackson T. C. Kochanek P. M. (2017). Adenosine production by brain cells. *J. Neurochem.* 141 676–693. 10.1111/jnc.14018 28294336 PMC5429183

[B89] Jacobson K. A. Balasubramanian R. Deflorian F. Gao Z. G. (2012). G protein-coupled adenosine (P1) and P2Y receptors: ligand design and receptor interactions. *Purinergic Signal.* 8 419–436. 10.1007/s11302-012-9294-9297 22371149 PMC3360101

[B90] Johnson R. A. Mitchell G. S. (2013). Common mechanisms of compensatory respiratory plasticity in spinal neurological disorders. *Respir. Physiol. Neurobiol.* 189 419–428. 10.1016/j.resp.2013.05.025 23727226 PMC3812344

[B91] Johnson S. M. Randhawa K. S. Baker T. L. Watters J. J. (2019). Respiratory frequency plasticity during development. *Respir. Physiol. Neurobiol.* 266 54–65. 10.1016/j.resp.2019.04.014 31055188 PMC6561787

[B92] Kawai A. Okada Y. Muckenhoff K. Scheid P. (1995). Theophylline and hypoxic ventilatory response in the rat isolated brainstem-spinal cord. *Respir. Physiol.* 100 25–32. 10.1016/0034-5687(94)00124-i 7604181

[B93] Kawamura M. Gachet C. Inoue K. Kato F. (2004). Direct excitation of inhibitory interneurons by extracellular ATP mediated by P2Y1 receptors in the hippocampal slice. *J. Neurosci.* 24 10835–10845. 10.1523/jneurosci.3028-04.2004 15574734 PMC6730213

[B94] King A. E. Ackley M. A. Cass C. E. Young J. D. Baldwin S. A. (2006). Nucleoside transporters: from scavengers to novel therapeutic targets. *Trends Pharmacol. Sci.* 27 416–425. 10.1016/j.tips.2006.06.004 16820221

[B95] Kleinfeld D. Deschenes M. Wang F. Moore J. D. (2014a). More than a rhythm of life: breathing as a binder of orofacial sensation. *Nat. Neurosci.* 17 647–651. 10.1038/nn.3693 24762718 PMC4140942

[B96] Kleinfeld D. Moore J. D. Wang F. Deschenes M. (2014b). The brainstem oscillator for whisking and the case for breathing as the master clock for orofacial motor actions. *Cold Spring Harb Symp. Quant. Biol.* 79 29–39. 10.1101/sqb.2014.79.024794 25876629 PMC4924579

[B97] Klyuch B. P. Dale N. Wall M. J. (2012). Receptor-mediated modulation of activity-dependent adenosine release in rat cerebellum. *Neuropharmacology* 62 815–824. 10.1016/j.neuropharm.2011.09.007 21933676

[B98] Klyuch B. P. Richardson M. J. Dale N. Wall M. J. (2011). The dynamics of single spike-evoked adenosine release in the cerebellum. *J. Physiol.* 589(Pt 2), 283–295. 10.1113/jphysiol.2010.198986 21078589 PMC3043533

[B99] Kobayashi K. Lemke R. P. Greer J. J. (2001). Development of fetal breathing movements in the rat. *J. Appl. Physiol.* 91 316–320. 11408446 10.1152/jappl.2001.91.1.316

[B100] Koles L. Furst S. Illes P. (2005). P2X and P2Y receptors as possible targets of therapeutic manipulations in CNS illnesses. *Drug News Perspect.* 18 85–101. 15883618 10.1358/dnp.2005.18.2.886479

[B101] Koos B. J. Chau A. Matsuura M. Punla O. Kruger L. (1998). Thalamic locus mediates hypoxic inhibition of breathing in fetal sheep. *J. Neurophysiol.* 79 2383–2393. 10.1152/jn.1998.79.5.2383 9582214

[B102] Koos B. J. Chau A. Matsuura M. Punla O. Kruger L. (2000). Thalamic lesions dissociate breathing inhibition by hypoxia and adenosine in fetal sheep. *Am. J. Physiol. Regul. Integr Comp. Physiol.* 278 R831–R837. 10.1152/ajpregu.2000.278.4.R831 10749769

[B103] Koos B. J. Kawasaki Y. Kim Y.-H. Bohorquez F. (2005). Adenosine A2A-receptor blockade abolishes the roll-off respiratory response to hypoxia in awake lambs. *Am. J. Physiol. Regul. Integr. Comp. Physiol.* 288 R1185–R1194. 15618344 10.1152/ajpregu.00723.2004

[B104] Koos B. J. Kruger L. Murray T. F. (1997). Source of extracellular brain adenosine during hypoxia in fetal sheep. *Brain Res.* 778 439–442. 10.1016/s0006-8993(97)01207-9 9459565

[B105] Koos B. J. Maeda T. (2001). Adenosine A(2A) receptors mediate cardiovascular responses to hypoxia in fetal sheep. *Am. J. Physiol. Heart Circ. Physiol.* 280 H83–H89. 10.1152/ajpheart.2001.280.1.H83 11123221

[B106] Koos B. J. Maeda T. Jan C. (2001). Adenosine A(1) and A(2A) receptors modulate sleep state and breathing in fetal sheep. *J. Appl. Physiol.* 91 343–350. 10.1152/jappl.2001.91.1.343 11408450

[B107] Koos B. J. Matsuda K. (1990). Fetal breathing, sleep state and cardiovascular responses to adenosine in sheep. *J. Appl. Physiol.* 68 489–495. 10.1152/jappl.1990.68.2.489 2108117

[B108] Kowaluk E. A. Jarvis M. F. (2000). Therapeutic potential of adenosine kinase inhibitors. *Expert Opin. Investig. Drugs* 9 551–564. 10.1517/13543784.9.3.551 11060695

[B109] Kull B. Svenningsson P. Fredholm B. B. (2000). Adenosine A(2A) receptors are colocalized with and activate g(olf) in rat striatum. *Mol. Pharmacol.* 58 771–777. 10.1124/mol.58.4.771 10999947

[B110] Lagercrantz H. Yamamoto Y. Fredholm B. B. Prabhakar N. R. Euler C. (1984). Adenosine analogues depress ventilation in rabbit neonates. *Paediatr. Res.* 18 387–390. 10.1203/00006450-198404000-00018 6326038

[B111] Lahiri S. Mitchell C. H. Reigada D. Ray A. Cherniack N. S. (2007). Purines, the carotid body and respiration. *Respir. Physiol. Neurobiol.* 157 123–129. 10.1016/j.resp.2007.02.015 17383945 PMC1975770

[B112] Langer D. Hammer K. Koszalka P. Schrader J. Robson S. Zimmermann H. (2008). Distribution of ectonucleotidases in the rodent brain revisited. *Cell Tissue Res.* 334 199–217. 10.1007/s00441-008-0681-x 18843508

[B113] Latini S. Pedata F. (2001). Adenosine in the central nervous system: release mechanisms and extracellular concentrations. *J. Neurochem.* 79 463–484. 10.1046/j.1471-4159.2001.00607.x 11701750

[B114] Leonard E. M. Salman S. Nurse C. A. (2018). Sensory processing and integration at the carotid body tripartite synapse: neurotransmitter functions and effects of chronic hypoxia. *Front. Physiol.* 9:225. 10.3389/fphys.2018.00225 29615922 PMC5864924

[B115] Leung R. S. Bradley T. D. (2001). Sleep apnea and cardiovascular disease. *Am. J. Respir. Crit. Care Med.* 164 2147–2165. 10.1164/ajrccm.164.12.2107045 11751180

[B116] Li P. Janczewski W. A. Yackle K. Kam K. Pagliardini S. Krasnow M. A. (2016). The peptidergic control circuit for sighing. *Nature* 530 293–297. 10.1038/nature16964 26855425 PMC4852886

[B117] Lietsche J. Imran I. Klein J. (2016). Extracellular levels of ATP and acetylcholine during lithium-pilocarpine induced status epilepticus in rats. *Neurosci. Lett.* 611 69–73. 10.1016/j.neulet.2015.11.028 26610905

[B118] Linden J. (1991). Structure and function of A1 adenosine receptors. *FASEB J.* 5 2668–2676. 10.1096/fasebj.5.12.1916091 1916091

[B119] Linden J. Tucker A. L. Lynch K. R. (1991). Molecular cloning of adenosine A1 and A2 receptors. *Trends Pharmacol. Sci.* 12 326–328. 10.1016/0165-6147(91)90589-k 1949201

[B120] Lloyd H. G. Fredholm B. B. (1995). Involvement of adenosine deaminase and adenosine kinase in regulating extracellular adenosine concentration in rat hippocampal slices. *Neurochem. Int.* 26 387–395. 10.1016/0197-0186(94)00144-j 7633332

[B121] Lloyd H. G. Lindstrom K. Fredholm B. B. (1993). Intracellular formation and release of adenosine from rat hippocampal slices evoked by electrical stimulation or energy depletion. *Neurochem. Int.* 23 173–185. 10.1016/0197-0186(93)90095-m 8369741

[B122] Lopes J. P. Pliassova A. Cunha R. A. (2019). The physiological effects of caffeine on synaptic transmission and plasticity in the mouse hippocampus selectively depend on adenosine A1 and A2A receptors. *Biochem. Pharmacol.* 166 313–321. 10.1016/j.bcp.2019.06.008 31199895

[B123] Lopes L. V. Cunha R. A. Kull B. Fredholm B. B. Ribeiro J. A. (2002). Adenosine A(2A) receptor facilitation of hippocampal synaptic transmission is dependent on tonic A(1) receptor inhibition. *Neuroscience* 112 319–329. 10.1016/s0306-4522(02)00080-5 12044450

[B124] Lopes L. V. Cunha R. A. Ribeiro J. A. (1999). Cross talk between A(1) and A(2A) adenosine receptors in the hippocampus and cortex of young adult and old rats. *J. Neurophysiol.* 82 3196–3203. 10.1152/jn.1999.82.6.3196 10601453

[B125] Lopes L. V. Rebola N. Pinheiro P. C. Richardson P. J. Oliveira C. R. Cunha R. A. (2003). Adenosine A3 receptors are located in neurons of the rat hippocampus. *Neuroreport* 14 1645–1648. 10.1097/01.wnr.0000088406.04452.44 14502093

[B126] Lorier A. R. Huxtable A. G. Robinson D. M. Lipski J. Housley G. D. Funk G. D. (2007). P2Y1 receptor modulation of the pre-Botzinger complex inspiratory rhythm generating network in vitro. *J. Neurosci.* 27 993–1005. 10.1523/jneurosci.3948-06.2007 17267553 PMC6673186

[B127] Lorier A. R. Lipski J. Housley G. D. Greer J. J. Funk G. D. (2008). ATP sensitivity of preBotzinger complex neurones in neonatal rat in vitro: mechanism underlying a P2 receptor-mediated increase in inspiratory frequency. *J. Physiol.* 586 1429–1446. 10.1113/jphysiol.2007.143024 18174215 PMC2375674

[B128] Mahan L. C. McVittie L. D. Smyk-Randall E. M. Nakata H. Monsma F. J. Jr. Gerfen C. R. (1991). Cloning and expression of an A1 adenosine receptor from rat brain. *Mol. Pharmacol.* 40 1–7. 1857334

[B129] Marina N. Turovsky E. Christie I. N. Hosford P. S. Hadjihambi A. Korsak A. (2017). Brain metabolic sensing and metabolic signaling at the level of an astrocyte. *Glia* 66 1185–1199. 10.1002/glia.23283 29274121 PMC5947829

[B130] Martin E. D. Fernandez M. Perea G. Pascual O. Haydon P. G. Araque A. (2007). Adenosine released by astrocytes contributes to hypoxia-induced modulation of synaptic transmission. *Glia* 55 36–45. 10.1002/glia.20431 17004232

[B131] Martin R. J. Abu-Shaweesh J. M. (2005). Control of breathing and neonatal apnea. *Biol. Neonate* 87 288–295. 10.1159/000084876 15985751

[B132] Mayer C. A. Haxhiu M. A. Martin R. J. Wilson C. G. (2006). Adenosine A2A receptors mediate GABAergic inhibition of respiration in immature rats. *J. Appl. Physiol.* 100 91–97. 10.1152/japplphysiol.00459.2005 16141383

[B133] McPherson C. Neil J. J. Tjoeng T. H. Pineda R. Inder T. E. (2015). A pilot randomized trial of high-dose caffeine therapy in preterm infants. *Pediatr. Res.* 78 198–204. 10.1038/pr.2015.72 25856169 PMC4928641

[B134] Meghji P. Tuttle J. B. Rubio R. (1989). Adenosine formation and release by embryonic chick neurons and glia in cell culture. *J. Neurochem.* 53 1852–1860. 10.1111/j.1471-4159.1989.tb09252.x 2553868

[B135] Mironov S. L. Langohr K. Richter D. W. (1999). A1 adenosine receptors modulate respiratory activity of the neonatal mouse via the cAMP-mediated signaling pathway. *J. Neurophysiol.* 81 247–255. 10.1152/jn.1999.81.1.247 9914285

[B136] Montandon G. Horner R. L. Kinkead R. Bairam A. (2009). Caffeine in the neonatal period induces long-lasting changes in sleep and breathing in adult rats. *J. Physiol.* 587(Pt 22), 5493–5507. 10.1113/jphysiol.2009.171918 19770189 PMC2793879

[B137] Mortola J. P. (1996). “Ventilatory responses to hypoxia in mammals,” in *Tissue Oxygen Deprivation*, eds Haddad G. G. Lister G. (New York, NY: Marcel Dekker), 433–477.

[B138] Moss I. R. Inman J. G. (1989). Neurochemicals and respiratory control during development. *J. Appl. Physiol.* 67 1–13. 10.1152/jappl.1989.67.1.1 2569452

[B139] Moss R. M. (2000). Respiratory responses to single and episodic hypoxia during development: mechanisms of adaptation. *Respir. Physiol.* 121 185–197. 10.1016/s0034-5687(00)00127-4 10963774

[B140] Mouro F. M. Rombo D. M. Dias R. B. Ribeiro J. A. Sebastiao A. M. (2018). Adenosine A2A receptors facilitate synaptic NMDA currents in CA1 pyramidal neurons. *Br. J. Pharmacol.* 175 4386–4397. 10.1111/bph.14497 30220081 PMC6240125

[B141] Nurse C. A. Leonard E. M. Salman S. (2018). Role of glial-like type II cells as paracrine modulators of carotid body chemoreception. *Physiol. Genomics* 50 255–262. 10.1152/physiolgenomics.00142.2017 29521602 PMC5966807

[B142] Pacak K. Gendron F. P. Benrezzak O. Krugh B. W. Kong Q. Weisman G. A. (2002). Purine signaling and potential new therapeutic approach: possible outcomes of NTPDase inhibition. *Curr. Drug Targets* 3 229–245. 12041737 10.2174/1389450023347713

[B143] Pak M. A. Haas H. L. Decking U. K. Schrader J. (1994). Inhibition of adenosine kinase increases endogenous adenosine and depresses neuronal activity in hippocampal slices. *Neuropharmacology* 33 1049–1053. 10.1016/0028-3908(94)90142-2 7838317

[B144] Parkinson F. E. Damaraju V. L. Graham K. Yao S. Y. Baldwin S. A. Cass C. E. (2011). Molecular biology of nucleoside transporters and their distributions and functions in the brain. *Curr. Top. Med. Chem.* 11 948–972. 10.2174/156802611795347582 21401500

[B145] Parkinson F. E. Xiong W. Zamzow C. R. (2005). Astrocytes and neurons: different roles in regulating adenosine levels. *Neurol. Res.* 27 153–160. 10.1179/016164105X21878 15829178

[B146] Pascual O. Casper K. B. Kubera C. Zhang J. Revilla-Sanchez R. Sul J. Y. (2005). Astrocytic purinergic signaling coordinates synaptic networks. *Science* 310 113–116. 10.1126/science.1116916 16210541

[B147] Pearson T. Currie A. J. Etherington L. A. Gadalla A. E. Damian K. Llaudet E. (2003). Plasticity of purine release during cerebral ischemia: clinical implications? *J. Cell Mol. Med.* 7 362–375. 10.1111/j.1582-4934.2003.tb00239.x 14754505 PMC6740112

[B148] Pedata F. Corsi C. Melani A. Bordoni F. Latini S. (2001). Adenosine extracellular brain concentrations and role of A2A receptors in ischemia. *Ann. N. Y. Acad. Sci.* 939 74–84. 10.1111/j.1749-6632.2001.tb03614.x 11462806

[B149] Poalillo P. Picone S. (2013). Apnea of prematurity. *J. Ped. Neon. Indiv. Med*. 2 e020213.

[B150] Punjabi N. M. (2008). The epidemiology of adult obstructive sleep apnea. *Proc. Am. Thorac. Soc.* 5 136–143. 10.1513/pats.200709-155MG 18250205 PMC2645248

[B151] Rajani R. Zhang Y. Jalubula V. Rancic V. SheikhBahaei S. Zwicker J. (2017). Release of ATP by preBötzinger complex astrocytes contributes to the hypoxic ventilatory response via a Ca2+-dependent P2Y1 receptor mechanism. *J. Physiol.* 596 3245–3269. 10.1113/jp274727 28678385 PMC6068109

[B152] Rajani V. Zhang Y. Revill A. L. Funk G. D. (2016). The role of P2Y1 receptor signaling in central respiratory control. *Respir. Physiol. Neurobiol.* 226 3–10. 10.1016/j.resp.2015.10.003 26476057

[B153] Rau A. R. Ariwodola O. J. Weiner J. L. (2015). Postsynaptic adenosine A2A receptors modulate intrinsic excitability of pyramidal cells in the rat basolateral amygdala. *Int. J. Neuropsychopharmacol.* 18:yv017. 10.1093/ijnp/pyv017 25716780 PMC4438553

[B154] Rebola N. Canas P. M. Oliveira C. R. Cunha R. A. (2005). Different synaptic and subsynaptic localization of adenosine A2A receptors in the hippocampus and striatum of the rat. *Neuroscience* 132 893–903. 10.1016/j.neuroscience.2005.01.014 15857695

[B155] Rebola N. Lujan R. Cunha R. A. Mulle C. (2008). Adenosine A2A receptors are essential for long-term potentiation of NMDA-EPSCs at hippocampal mossy fiber synapses. *Neuron* 57 121–134. 10.1016/j.neuron.2007.11.023 18184569

[B156] Rebola N. Pinheiro P. C. Oliveira C. R. Malva J. O. Cunha R. A. (2003). Subcellular localization of adenosine A(1) receptors in nerve terminals and synapses of the rat hippocampus. *Brain Res.* 987 49–58. 10.1016/s0006-8993(03)03247-5 14499945

[B157] Reppert S. M. Weaver D. R. Stehle D. R. Rivkees S. A. (1991). Molecular cloning and characterization of a rat A1-adenosine receptor that is widely expressed in brain and spinal cord. *Mol. Endocrinol.* 5 1037–1048. 10.1210/mend-5-8-1037 1658635

[B158] Richerson G. B. Boison D. Faingold C. L. Ryvlin P. (2016). From unwitnessed fatality to witnessed rescue: Pharmacologic intervention in sudden unexpected death in epilepsy. *Epilepsia* 57(Suppl. 1), 35–45. 10.1111/epi.13236 26749015 PMC4890608

[B159] Rodrigues R. J. Almeida T. Richardson P. J. Oliveira C. R. Cunha R. A. (2005). Dual presynaptic control by ATP of glutamate release via facilitatory P2X1, P2X2/3, and P2X3 and inhibitory P2Y1, P2Y2, and/or P2Y4 receptors in the rat hippocampus. *J. Neurosci.* 25 6286–6295. 10.1523/jneurosci.0628-05.2005 16000618 PMC6725280

[B160] Rosin D. L. Robeva A. Woodard R. L. Guyenet P. G. Linden J. (1998). Immunohistochemical localization of adenosine A2A receptors in the rat central nervous system. *J. Comp. Neurol.* 401 163–186. 10.1002/(sici)1096-9861(19981116)401:2<163::aid-cne2>3.0.co;2-d 9822147

[B161] Runold M. Lagercrantz H. Fredholm B. B. (1986). Ventilatory effect of an adenosine analogue in unanaesthetized rabbits during development. *J. Appl. Physiol.* 61 255–259. 10.1152/jappl.1986.61.1.255 3015860

[B162] Runold M. Lagercrantz H. Prabhakar N. R. Fredholm B. B. (1989). Role of adenosine in hypoxic ventilatory depression. *J. Appl. Physiol.* 67 541–546. 10.1152/jappl.1989.67.2.541 2793655

[B163] Scanziani M. Capogna M. Gahwiler B. H. Thompson S. M. (1992). Presynaptic inhibition of miniature excitatory synaptic currents by baclofen and adenosine in the hippocampus. *Neuron* 9 919–927. 10.1016/0896-6273(92)90244-8 1358131

[B164] Schmidt B. Anderson P. J. Doyle L. W. Dewey D. Grunau R. E. Asztalos E. V. (2012). Survival without disability to age 5 years after neonatal caffeine therapy for apnea of prematurity. *JAMA* 307 275–282. 10.1001/jama.2011.2024 22253394

[B165] Schmidt B. Roberts R. S. Davis P. Doyle L. W. Barrington K. J. Ohlsson A. (2007). Long-term effects of caffeine therapy for apnea of prematurity. *N Engl. J. Med.* 357 1893–1902. 10.1056/NEJMoa073679 17989382

[B166] Schmidt C. Bellingham M. C. Richter D. W. (1995). Adenosinergic modulation of respiratory neurones and hypoxic responses in the anaesthetized cat. *J. Physiol.* 483(Pt 3), 769–781. 10.1113/jphysiol.1995.sp020621 7776257 PMC1157817

[B167] Scholz K. P. Miller R. J. (1992). Inhibition of quantal transmitter release in the absence of calcium influx by a G protein-linked adenosine receptor at hippocampal synapses. *Neuron* 8 1139–1150. 10.1016/0896-6273(92)90134-y 1351733

[B168] Schulte G. Fredholm B. B. (2003). Signalling from adenosine receptors to mitogen-activated protein kinases. *Cell Signal.* 15 813–827. 10.1016/s0898-6568(03)00058-5 12834807

[B169] Sebastiao A. M. Ribeiro J. A. (2009). Adenosine receptors and the central nervous system. *Handb. Exp. Pharmacol.* 193 471–534. 10.1007/978-3-540-89615-9_16 19639292

[B170] Sheikhbahaei S. Turovsky E. A. Hosford P. S. Hadjihambi A. Theparambil S. M. Liu B. (2018). Astrocytes modulate brainstem respiratory rhythm-generating circuits and determine exercise capacity. *Nat. Commun.* 9:370. 10.1038/s41467-017-02723-2726 29371650 PMC5785528

[B171] Shen H. Y. Li T. Boison D. (2010). A novel mouse model for sudden unexpected death in epilepsy (SUDEP): role of impaired adenosine clearance. *Epilepsia* 51 465–468. 10.1111/j.1528-1167.2009.02248.x 19674057 PMC2844921

[B172] Shrestha B. Jawa G. (2017). Caffeine citrate - is it a silver bullet in neonatology? *Pediatr. Neonatol.* 58 391–397. 10.1016/j.pedneo.2016.10.003 28446386

[B173] Silinsky E. M. (1984). On the mechanism by which adenosine receptor activation inhibits the release of acetylcholine from motor nerve endings. *J. Physiol.* 346 243–256. 10.1113/jphysiol.1984.sp015019 6321717 PMC1199496

[B174] Sillanpaa M. Shinnar S. (2010). Long-term mortality in childhood-onset epilepsy. *N. Engl. J. Med.* 363 2522–2529. 10.1056/NEJMoa0911610 21175314

[B175] Statistics Canada. (2014). *Birth Database (CANSIM table 102–4512).* Ottawa: Statistics Canada.

[B176] Stella L. Berrino L. Maione S. de Novellis V. Rossi F. (1993). Cardiovascular effects of adenosine and its analogs in anaesthetized rats. *Life Sci.* 53 755–763. 10.1016/0024-3205(93)90497-q 8355564

[B177] Svenningsson P. Hall H. Sedvall G. Fredholm B. B. (1997a). Distribution of adenosine receptors in the postmortem human brain: an extended autoradiographic study. *Synapse* 27 322–335. 10.1002/(sici)1098-2396(199712)27:4<322::aid-syn6>3.0.co;2-e 9372555

[B178] Svenningsson P. Le Moine C. Kull B. Sunahara R. Bloch B. Fredholm B. B. (1997b). Cellular expression of adenosine A2A receptor messenger RNA in the rat central nervous system with special reference to dopamine innervated areas. *Neuroscience* 80 1171–1185. 10.1016/s0306-4522(97)00180-2 9284069

[B179] Takahashi M. Fujita M. Asai N. Saki M. Mori A. (2018). Safety and effectiveness of istradefylline in patients with Parkinson’s disease: interim analysis of a post-marketing surveillance study in Japan. *Expert Opin. Pharmacother.* 19 1635–1642. 10.1080/14656566.2018.1518433 30281377

[B180] Teppema L. J. (2018a). CrossTalk opposing view: the hypoxic ventilatory response does not include a central, excitatory hypoxia sensing component. *J. Physiol.* 596 2939–2941. 10.1113/JP275708 29947097 PMC6068226

[B181] Teppema L. J. (2018b). Rebuttal from Luc J. Teppema. *J. Physiol.* 596 2945. 10.1113/JP276281 29947023 PMC6068114

[B182] Teppema L. J. Dahan A. (2010). The ventilatory response to hypoxia in mammals: mechanisms, measurement, and analysis. *Physiol. Rev.* 90 675–754. 10.1152/physrev.00012.2009 20393196

[B183] Tetzlaff W. Schubert P. Kreutzberg G. W. (1987). Synaptic and extrasynaptic localization of adenosine binding sites in the rat hippocampus. *Neuroscience* 21 869–875. 10.1016/0306-4522(87)90043-1 3627439

[B184] Thauerer B. Zur Nedden S. Baier-Bitterlich G. (2012). Purine nucleosides: endogenous neuroprotectants in hypoxic brain. *J. Neurochem.* 121 329–342. 10.1111/j.1471-4159.2012.07692.x 22335456 PMC3499684

[B185] Theofilas P. Brar S. Stewart K. A. Shen H. Y. Sandau U. S. Poulsen D. (2011). Adenosine kinase as a target for therapeutic antisense strategies in epilepsy. *Epilepsia* 52 589–601. 10.1111/j.1528-1167.2010.02947.x 21275977 PMC3075862

[B186] Thomas T. Spyer K. M. (2000). ATP as a mediator of mammalian central CO2 chemoreception. *J. Physiol.* 523(Pt 2), 441–447. 10.1111/j.1469-7793.2000.00441.x 10699087 PMC2269817

[B187] Tomaselli B. Nedden S. Z. Podhraski V. Baier-Bitterlich G. (2008). p42/44 MAPK is an essential effector for purine nucleoside-mediated neuroprotection of hypoxic PC12 cells and primary cerebellar granule neurons. *Mol. Cell Neurosci.* 38 559–568. 10.1016/j.mcn.2008.05.004 18585057

[B188] Tronche F. Kellendonk C. Kretz O. Gass P. Anlag K. Orban P. C. (1999). Disruption of the glucocorticoid receptor gene in the nervous system results in reduced anxiety. *Nat. Genet.* 23 99–103. 10.1038/12703 10471508

[B189] van Calker D. Biber K. (2005). The role of glial adenosine receptors in neural resilience and the neurobiology of mood disorders. *Neurochem. Res.* 30 1205–1217. 10.1007/s11064-005-8792-8791 16341582

[B190] van Calker D. Muller M. Hamprecht B. (1979). Adenosine regulates via two different types of receptors, the accumulation of cyclic AMP in cultured brain cells. *J. Neurochem.* 33 999–1005. 10.1111/j.1471-4159.1979.tb05236.x 228008

[B191] Vandam R. J. Shields E. J. Kelty J. D. (2008). Rhythm generation by the pre-Botzinger complex in medullary slice and island preparations: effects of adenosine A(1) receptor activation. *BMC Neurosci.* 9:95. 10.1186/1471-2202-9-95 18826652 PMC2567986

[B192] Vilella L. Lacuey N. Hampson J. P. Rani M. R. S. Sainju R. K. Friedman D. (2018). Postconvulsive central apnea as a biomarker for sudden unexpected death in epilepsy (SUDEP). *Neurology* 92 e171–e182. 10.1212/WNL.0000000000006785 30568003 PMC6340388

[B193] Volonte C. D’Ambrosi N. (2009). Membrane compartments and purinergic signalling: the purinome, a complex interplay among ligands, degrading enzymes, receptors and transporters. *FEBS J.* 276 318–329. 10.1111/j.1742-4658.2008.06793.x 19076212

[B194] Wall M. Dale N. (2008). Activity-dependent release of adenosine: a critical re-evaluation of mechanism. *Curr. Neuropharmacol.* 6 329–337. 10.2174/157015908787386087 19587854 PMC2701281

[B195] Wall M. J. Dale N. (2007). Auto-inhibition of rat parallel fibre-Purkinje cell synapses by activity-dependent adenosine release. *J. Physiol.* 581(Pt 2), 553–565. 10.1113/jphysiol.2006.126417 17347275 PMC2075183

[B196] Wang J.-L. Wu Z.-H. Pan B.-X. Li J. (2005). Adenosine A1 receptors modulate the discharge activities of inspiratory and biphasic expiratory neurons in the meial regions o the nucleus retrofacialis of neonatal rat in vitro. *Neurosci. Lett.* 379 27–31. 10.1016/j.neulet.2004.12.042 15814193

[B197] Wei C. J. Li W. Chen J. F. (2011). Normal and abnormal functions of adenosine receptors in the central nervous system revealed by genetic knockout studies. *Biochim. Biophys. Acta* 1808 1358–1379. 10.1016/j.bbamem.2010.12.018 21185258

[B198] Wessberg P. Hedner J. Hedner T. Persson B. Jonason J. (1984). Adenosine mechanisms in the regulation of breathing in the rat. *Eur. J. Pharmacol.* 106 59–67. 10.1016/0014-2999(84)90678-2 6099272

[B199] Williams-Karnesky R. L. Sandau U. S. Lusardi T. A. Lytle N. K. Farrell J. M. Pritchard E. M. (2013). Epigenetic changes induced by adenosine augmentation therapy prevent epileptogenesis. *J. Clin. Invest.* 123 3552–3563. 10.1172/JCI65636 23863710 PMC3726154

[B200] Wilson C. G. Martin R. J. Jaber M. Abu-Shaweesh J. Jafri A. Haxhiu M. A. (2004). Adenosine A2A receptors interact with GABAergic pathways to modulate respiration in neonatal piglets. *Respir. Physiol. Neurobiol.* 141 201–211. 10.1016/j.resp.2004.04.012 15239970

[B201] Wu L. G. Saggau P. (1994). Adenosine inhibits evoked synaptic transmission primarily by reducing presynaptic calcium influx in area CA1 of hippocampus. *Neuron* 12 1139–1148. 10.1016/0896-6273(94)90321-2 8185949

[B202] Yamamoto M. Nishimura M. Kobayashi S. Akiyama Y. Miyamoto K. Kawakami Y. (1994). Role of endogenous adenosine in hypoxic ventilatory response in humans: a study with dipyridamole. *J. Appl. Physiol.* 76 196–203. 10.1152/jappl.1994.76.1.196 8175505

[B203] Yan S. Laferriere A. Zhang C. Moss I. R. (1995). Microdialyzed adenosine in nucleus tractus solitarii and ventilatory response to hypoxia in piglets. *J. Appl. Physiol.* 79 405–410. 10.1152/jappl.1995.79.2.405 7592195

[B204] Yawo H. Chuhma N. (1993). Preferential inhibition of omega-conotoxin-sensitive presynaptic Ca2+ channels by adenosine autoreceptors. *Nature* 365 256–258. 10.1038/365256a0 8396730

[B205] Young J. D. Yao S. Y. Baldwin J. M. Cass C. E. Baldwin S. A. (2013). The human concentrative and equilibrative nucleoside transporter families. SLC28 and SLC29. *Mol. Aspects Med.* 34 529–547. 10.1016/j.mam.2012.05.007 23506887

[B206] Young T. Palta M. Dempsey J. Skatrud J. Weber S. Badr S. (1993). The occurrence of sleep-disordered breathing among middle-aged adults. *N. Engl. J. Med.* 328 1230–1235. 10.1056/NEJM199304293281704 8464434

[B207] Young T. Peppard P. (2000). Sleep-disordered breathing and cardiovascular disease: epidemiologic evidence for a relationship. *Sleep* 23(Suppl. 4), S122–S126. 10893084

[B208] Zhang D. Xiong W. Chu S. Sun C. Albensi B. C. Parkinson F. E. (2012). Inhibition of hippocampal synaptic activity by ATP, hypoxia or oxygen-glucose deprivation does not require CD73. *PLoS One* 7:e39772. 10.1371/journal.pone.0039772 22761898 PMC3382561

[B209] Zwicker J. D. Rajani V. Hahn L. B. Funk G. D. (2011). Purinergic modulation of preBotzinger complex inspiratory rhythm in rodents: the interaction between ATP and adenosine. *J. Physiol.* 589(Pt 18), 4583–4600. 10.1113/jphysiol.2011.210930 21788352 PMC3208226

[B210] Zwicker J. D. Zhang Y. Ren J. Hutchinson M. R. Rice K. C. Watkins L. R. (2014). Glial TLR4 signaling does not contribute to opioid-induced depression of respiration. *J. Appl. Physiol.* 117 857–868. 10.1152/japplphysiol.00534.2014 25103966 PMC4199989

